# Large Scale Conversion of Trilobolide into the Payload of Mipsagargin: 8-*O*-(12-Aminododecanoyl)-8-*O*-Debutanoylthapsigargin

**DOI:** 10.3390/biom10121640

**Published:** 2020-12-05

**Authors:** Tomáš Zimmermann, Pavel Drašar, Silvie Rimpelová, Søren Brøgger Christensen, Vladimir A. Khripach, Michal Jurášek

**Affiliations:** 1 Department of Chemistry of Natural Compounds, University of Chemistry and Technology Prague, Technická 5, 166 28 Prague, Czech Republic; tomas.zimmermann@vscht.cz (T.Z.); pavel.drasar@vscht.cz (P.D.); 2 Department of Biochemistry and Microbiology, University of Chemistry and Technology Prague, Technická 5, 166 28 Prague, Czech Republic; silvie.rimpelova@vscht.cz; 3 Department of Drug Design and Pharmacology, University of Copenhagen, Jagtvej 162, DK-2100 Copenhagen, Denmark; soren.christensen@sund.ku.dk; 4 Institute of Bioorganic Chemistry, National Academy of Sciences of Belarus, Kuprevich St. 5, 220141 Minsk, Belarus; khripach@iboch.by

**Keywords:** chemical synthesis, extraction, mipsagargin, trilobolide isolation from fruits, optimization and scale-up, *Laser trilobum* cultivation, sarco/endoplasmic reticulum calcium ATPase (SERCA), sesquiterpene lactones, thapsigargin, trilobolide, 8-*O*-(12-aminododecanoyl)-8-*O*-debutanoylthapsigargin

## Abstract

In spite of the impressing cytotoxicity of thapsigargin (**Tg**), this compound cannot be used as a chemotherapeutic drug because of general toxicity, causing unacceptable side effects. Instead, a prodrug targeted towards tumors, mipsagargin, was brought into clinical trials. What substantially reduces the clinical potential is the limited access to Tg and its derivatives and cost-inefficient syntheses with unacceptably low yields. *Laser trilobum*, which contains a structurally related sesquiterpene lactone, trilobolide (**Tb**), is successfully cultivated. Here, we report scalable isolation of **Tb** from *L. trilobum* and a transformation of **Tb** to 8-*O*-(12-aminododecanoyl)-8-*O*-debutanoylthapsigargin in seven steps. The use of cultivated *L. trilobum* offers an unlimited source of the active principle in mipsagargin.

## 1. Introduction

Substances able to induce endoplasmic reticulum (ER) stress are suitable tools in cancer research and therapy. Thapsigargin (**Tg**), which is isolated from *Thapsia garganica* L. (Apiaceae) [1], induces apoptosis in all types of cancer cell lines [2,3,4] regardless of the phase of the cell cycle [5]. This feature overcomes the drawback of chemotherapeutics such as paclitaxel and docetaxel, currently used in clinics as first- and second-line therapies, which act as mitotic poisons (reviewed in [6]). Their utilization is, therefore, limited to the treatment of rapidly growing tumors [7]. An urgent need exists for the development of new chemotherapeutics with enhanced efficiency and selectivity for the treatment of slow-growing tumors, such as hepatocellular carcinoma and prostate carcinoma [8]. Since **Tg** kills all cells irrespective of their phase in the cell cycle, this compound could be an attractive anticancer drug if a drug targeted towards tumors is developed.

**Tg** potently inhibits all members of the enzyme family sarco-endoplasmic reticulum calcium ATPase (SERCA; reviewed in [9]). Inhibition of SERCA provokes depletion of Ca^2+^ in the endoplasmic reticulum (ER) [10], leading to the unfolded protein response (UPR) [11]. Prolonged UPR causes mitochondrial damage, resulting in apoptosis [12]. 

**Figure 1:**
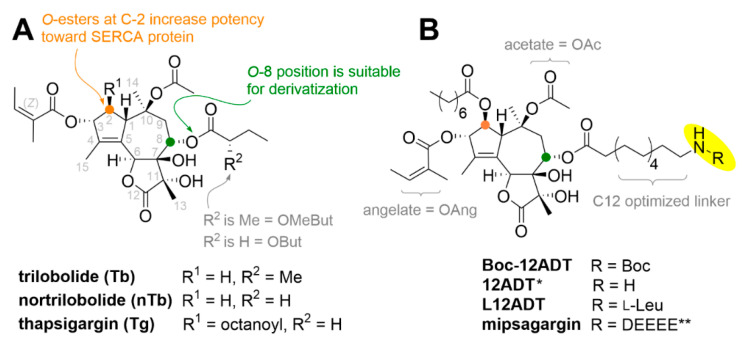
Molecular structures of thapsigargins (Panel **A**) and structures of researched thapsigargin (Tg) derivatives (Panel **B**). * 8-*O*-(12-aminododecanoyl)-8-*O*-debutanoyl-**Tg**; ** DEEEE = β-L-Asp-γ-(L-Glu)_4_-OH.

**Figure 2:**
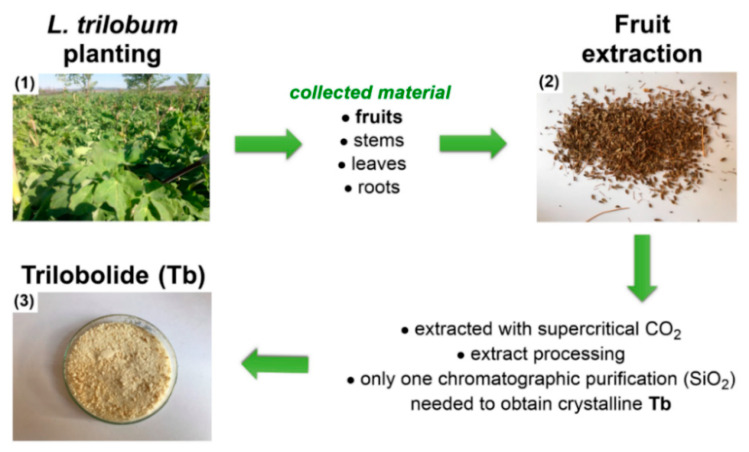
Optimized route for large scale **Tb** isolation, i.e., (**1**) cultivation of *L. trilobum* and plant harvesting, and (**2**) drying of fruits and gram-scale isolation of **Tb** (**3**).

**Tg** is distinguished from trilobolide (**Tb**) by the presence of an octanoate group at C-2 and a missing methyl group at the α-position in the *O*-8 side chain (Figure 1) [13,14,15]. **Tb** is isolated from *Laser trilobum* (L.) Borkh (Apiaceae). In English, *L. trilobum* is named horse caraway (Figure 2).

Due to the high systemic toxicity of **Tg**, a prodrug strategy for specific targeting of cancerous tissues was introduced [16]. The prodrugs were based on 8-*O*-(12-aminododecanoyl)-8-*O*-debutanoylthapsigargin (**12ADT**) [17]. By choosing a peptide, which is a substrate for an enzyme overexpressed on the surface of cancer cells, or an enzyme that is excreted by cancer cells, selectivity might be obtained. Three peptides that are fast cleaved by the enzymes that are characteristic of prostatic cancer cell lines are GKAFRRL, HSSKLQL, and βDγEγEγEγE. GKAFRRL is cleaved by human kallikrein-related peptidase 2 (KLK2; formerly human kallikrein 2 or hK2), HSSKLQL by prostate-specific antigen (PSA), and βDγEγEγEγE by prostate-specific membrane antigen (PSMA = glutamate carboxypeptidase II = *N*-acetyl-L-aspartyl-L-glutamate peptidase I) [18]. These peptides are cleaved only to a limited extent by other proteases present in the human body. The absence of an amino group prevents **Tg** from being conjugated to a peptide. The prodrug mipsagargin, consisting of 8-*O*-(12-aminododecanoyl)-8-*O*-debutanoulthapsigargin bound to peptide βDγEγEγEγE, was developed by Inspyr Therapeutics, formerly GenSpera [19,20] (Figure 1B), and advanced to clinical trials II as second-line therapy for the treatment of advanced hepatocellular carcinoma. The prodrug was able to decrease blood flow to tumors by 52% on average. However, unspecific toxicity prevented the use of higher doses of the prodrug, which might improve the therapy outcome. Consequently, the drug was not subjected to the third phase of clinical trials. Despite the polar peptide, mipsagargin most likely penetrates cell membranes, causing systemic toxicity in patients [21]. However, a novel and more complex strategy for specific **Tg** targeting has been introduced and evaluated for possible treatment of metastatic castration-resistant prostate carcinoma [22,23]. This method is based on the coupling of PSA-targeted **Tg** prodrug via a 2-fluoro-5-maleimidobenzamide linker to human serum albumin (HSA) [23]. Coupling to HSA offers favorable properties for the prodrug, such as long half-life in blood, prevention of nonselective permeation into benign cells [1,23], and accumulation in cancer tissue.

A successful outcome of the development of a new **Tg** prodrug for the treatment of cancer diseases will create a yearly demand of 1 metric ton. This supply cannot be met by the isolation of **Tg** from fruits of the wild population of *T. garganica*. *L. trilobum* is successfully cultivated in field conditions in the Czech Republic, and, therefore, unlimited plant material for large-scale **Tb** extraction can be obtained. In terms of total synthesis, firstly, the number of steps in the published methods for the total synthesis of **Tg** preclude their use for commercial production, and, secondly, the total yields are rather poor [24,25]. Another option is to take advantage of the published procedure for converting nortrilobolide (**nTb**) into **Tg** [26,27]. Here, we describe a procedure for transforming **Tb** into the cytotoxin in the prodrug mipsagargin, 8-*O*-(12-aminododecanoyl)-8-*O*-debutanlyothapsigargin in gram scale. In addition, a procedure for the isolation of **Tb** on the decagram scale is described.

## 2. Materials and Methods 

For thin-layer chromatography (TLC), aluminum silica gel sheets for detection in UV light (TLC silica gel 60 F254, Merck) were used. For extractions of plant material by supercritical CO_2_, a NATEX extractor (Ternitz, Austria) was used. For TLC visualization, a diluted solution of H_2_SO_4_ in MeOH was used and plates were heated. For column chromatography, 30–60 μm silica gel (ICN Biomedicals, Costa Mesa, USA) was used. NMR spectra were recorded by Agilent-MR DDR2 (Varian, Palo Alto, CA, USA). Chemical shifts are given as δ values. The Quadrupole LC/MS (ESI ionization) with an Infinity III LC system (Agilent Technologies, Santa Clara, USA) was used for LR-MS and HPLC analyses (C18 column: 100 mm; UV detection).

The following chemicals were purchased from TCI Europe (Zwijndrecht, Belgium): *N,N,N*-triethylamine—TEA (>99%), 4-dimethylaminopyridine—4-DMAP (>99%), ethyl-dimethylaminopropyl carbodiimide—EDC (>98%), angelic acid (>98%), 2,4,6-trichlorobenzoyl chloride (>98%), 12-aminododecanoic acid (>98%), di-*tert*-butyl decarbonate—Boc_2_O (>95%), trifluoroacetic acid—TFA (>99%). The following chemicals were purchased from Sigma-Aldrich (Prague, Czech Republic): chromium (VI)oxide—CrO_3_ (≥98%), zinc(II) chloride—ZnCl_2_ (≥98%), sodium borohydride (≥98%), and manganese (III) acetate dihydrate—Mn(OAc)_3_ · 2H_2_O (97%). The solvents for column chromatography and reactions were purchased from PENTA (Praha, Czech Republic) and were used without further distillation.

*L. trilobum* has been largely cultivated in fields in the Czech Republic (South Moravia region). A voucher specimen was deposited under the code 03013KBFR in the herbarium of the Department of Botany and Plant Physiology of the Faculty of Agrobiology, Food and Natural Resources of the Czech University of Life Sciences, Prague (Czech Republic), and at the University of Chemical Technology, Prague (Czech Republic).

### 2.1. Supercritical Carbon Dioxide Extraction of Trilobolide from L. Trilobum Fruits

Fruits of horse caraway *L. trilobum*, grown in a field in the South Moravia region, were collected manually and dried. The fruits (30 kg) were crushed and extracted by supercritical CO_2_ at 50 °C for 1 h at the flow rate of 600 kg of CO_2_ per hour. During the extraction process, two fractions were taken. The first fraction, taken for the initial 30 min, afforded 1.85 kg, and the second fraction, taken for the subsequent 30 min, afforded 1.09 kg. The larger first fraction, which contained more **Tb** (R_f_ = 0.4) than the second one (confirmed by TLC using hexanes–AcOEt (2:1, *v/v*) as an eluent), was divided into 9 portions of ca. 200 g. Each 200 g fraction was vigorously mixed with hexanes (400 mL), and, after 1 h, the insoluble portion was removed by filtering through a folded paper filter. The amorphous greasy matter on the filter (53 g) was suspended into MeOH (200 mL) and vigorously stirred for 1 h, and the mixture was filtered through a folded filter paper. The filtrate was concentrated to yield thick syrupy material (10 g). All nine portions of the syrupy material were combined and dissolved in CHCl_3_–MeOH (1:1, *v/v*) mixture. The solution was mixed with silica gel, and the solvent was evaporated. The powdery material was applied on a column of the same silica gel (length of 25 cm, a diameter of 6 cm), washed by hexanes (2 L), followed by DCM (10 L). Evaporation of the DCM eluate gave brown-yellow syrupy material (41 g) that contained traces of **Tb** and mostly lipophilic components (TLC using hexanes–AcOEt (2:1, *v/v*) as an eluent). Subsequently, the column was washed by a mixture of DCM-MeOH (1:1, *v/v*), affording 61.5 g of an oily residue after evaporation of the eluent. The eluate contained almost exclusively **Tb**. The oily residue (61.5 g) was dissolved in AcOEt (120 mL), hexanes (200 mL) were added, and the mixture was left overnight at 4 °C. The formed crystals were obtained on a sintered glass filter by filtration, yielding 40.7 g of **Tb** as colorless crystals (0.14% on the weight of the fruits). The NMR spectra of this product were identical to those previously obtained for **Tb** [15].

### 2.2. Scale-Up Synthesis of 8-O-(12-Aminododecanoyl)-8-O-Debutanoyl Thapsigargin (**12ADT**) from **Tb**

#### 2.2.1. Synthesis of 3-oxo-3-Desangeloyl Trilobolide (**1**)

**Tb** (5.25 g, 9.6 mmol), placed into a 0.5-L round-bottom flask, was dissolved in MeCN (250 mL) and CrO_3_ (670 g, 6.7 mmol), and aqueous 1 M HF (21 mL, 19.1 mmol) was added. The mixture was heated to 95 °C and stirred for 2.5 h. Gradual color change of the reaction mixture from red to green indicated the reduction of CrO_3_ to Cr_2_O_3_. After 2.5 h, the reaction was quenched by addition of a saturated NaHCO_3_ (20 mL) solution in water. After evaporation of MeCN, AcOEt (50 mL) was added and the mixture was filtered. The organic phase was washed with brine (2 × 50 mL). The combined water phases were once extracted with AcOEt (30 mL) and washed with brine (30 mL). The organic layer was dried over Na_2_SO_4_, filtered, and concentrated. Chromatography over a silica gel column (length of 24 cm, a diameter of 4 cm; hexanes–AcOEt (3:1, *v/v*) as an eluent) afforded ketone **1** as white powder (3.6 g, 8.2 mmol) at 82% yield. Unreacted starting **Tb** (275 mg, 1.01 mmol) was recovered. **R_f_** = 0.54 in hexanes–AcOEt (1:1, *v/v*) as an eluent. **^1^H NMR** (400 MHz, CDCl_3_) δ: 5.78 (s, 1H), 5.71 (t, J = 3.7 Hz, 1H), 4.82–4.75 (m, 1H), 3.34 (dd, J = 14.8, 3.7 Hz, 1H), 2.49–2.36 (m, 2H), 2.36–2.29 (m, 1H), 2.07 (dd, J = 14.7, 3.7 Hz, 1H), 1.98 (s, 3H), 1.94 (s, 3H), 1.76–1.58 (m, 3H), 1.52 (s, 3H), 1.21 (s, 3H), 1.13 (d, J = 7.0 Hz, 3H), 0.89 (t, J = 7.4 Hz, 3H); Figure S1A. **^13^C NMR** (100 MHz, CDCl_3_) δ: 207.22, 175.43, 174.81, 171.23, 159.45, 144.80, 85.13, 79.08, 78.64, 77.65, 66.26, 45.87, 41.37, 38.91, 36.53, 26.11, 22.30, 22.26, 16.27, 16.17, 11.62, 9.75; Figure S1B. **HMQC** is shown in Figure S1C. **HRMS-ESI**: for [C_22_H_30_O_9_ + Na]^+^ calculated *m/z*: 461.17820 Da, found *m/z*: 461.17824; Figure S1D.

#### 2.2.2. Synthesis of 3-Oxo-2-Octanoyl-3-Desangeloyl Trilobolide (**2**)

Ketone **1** (2.15 g, 4.9 mmol) was dissolved in mixture of benzene (250 mL) and octanoic acid (50 mL) in a 0.5-L round-bottom flask. The mixture was heated to 60 °C, and Mn(OAc)_3_ · 2H_2_O, (2.7 g, 10 mmol) was added. The temperature was raised to 120 °C. Water formed was removed by Dean–Stark apparatus. The mixture was then stirred for 6 h and cooled to RT (20 °C). The mixture was concentrated using a rotary evaporator and the obtained residue was dissolved in AcOEt (100 mL). The solution was washed with 2 M Na_2_CO_3_ (300 mL). Formed sodium octanoate salt was removed by filtration through filter paper. The resulting organic and water phases were poured into a separation funnel; the organic layer was collected and washed with brine (2 × 50 mL) and 2 M Na_2_CO_3_ (2 × 50 mL) to ensure of removal of the octanoic acid. The separated organic layer was dried over Na_2_SO_4_, filtered, and the solvents were evaporated under reduced pressure. Chromatography over a silica gel column (length of 30 cm, a diameter of 4 cm using hexanes–AcOEt (3:1, *v/v*) as an eluent) provided α-ketoester **2** (1.78 g; 3.1 mmol) as orange powder at 63% yield. Unreacted starting material **1** (560 mg, 1.3 mmol) was recovered. **R_f_** = 0.36 using hexanes–AcOEt (2:1, *v/v*) as an eluent. **^1^H NMR** (400 MHz, CDCl_3_) δ: 5.79 (s, 1H), 5.68 (t, J = 3.7 Hz, 1H), 5.18 (d, J = 3.3 Hz, 1H), 4.60 (d, J = 2.4 Hz, 1H), 3.27 (dd, J = 14.8, 3.7 Hz, 1H), 2.37–2.31 (m, 2H), 2.32–2.28 (m, 1H), 2.18 (dd, J = 14.7, 3.8 Hz, 1H), 1.99 (s, 3H), 1.93 (s, 3H), 1.64–1.55 (m, 2H), 1.47 (s, 3H), 1.38 (s, 3H), 1.35–1.21 (m, 10H), 1.13 (d, J = 7.0 Hz, 3H), 0.90 (t, J = 7.4 Hz, 3H), 0.86 (t, J = 5.8 Hz, 3H); Figure S2A. **^13^C NMR** (100 MHz, CDCl_3_) δ: 201.43, 175.50, 174.95, 172.93, 170.95, 156.60, 142.02, 83.93, 78.93, 78.49, 77.84, 73.06, 66.07, 51.61, 41.42, 38.67, 33.81, 31.61, 28.94, 28.91, 26.11, 24.70, 23.05, 22.58, 22.48, 16.22, 16.01, 14.06, 11.61, 10.24; Figure S2B. **HMQC** is displayed in Figure S2C. **HRMS-ESI**: for [C_30_H_44_O_11_ + Na]^+^ calculated *m/z*: 603.27768 Da, found *m/z*: 603.27795; Figure S2D.

#### 2.2.3. Synthesis of (3*S*)-Hydroxy-2-Octanoyl-3-Desangeloyl Trilobolide (**3**)

α-Ketoester **2** (1.78 g, 3.07 mmol), dissolved in Et_2_O (120 mL) under an argon atmosphere, was cooled to -20 °C. Freshly prepared Zn(BH_4_)_2_ (70 mL; 0.5 M in Et_2_O) was added dropwise via a syringe and the reaction was stirred for 3.5 h. The reaction was quenched by dropwise addition of H_2_O (20 mL). After addition of AcOEt (100 mL), the Et_2_O was evaporated at 500 mbar. The resulting mixture of AcOEt and H_2_O was filtered through filter paper to remove white sludge formed by Zn(BH_4_)_2_ decomposition. The resulting filtered phases were poured into a separator and the collected organic phase was washed with brine (2 × 100 mL). The organic layer was dried over Na_2_SO_4_, filtered, and the solvents were evaporated under reduced pressure. Chromatography over a silica gel column (length of 30 cm, a diameter of 4 cm; using hexanes–AcOEt (2:1, *v/v*) as an eluent) afforded α-hydroxy ester **3** as a white powder (1.41 g, 2.45 mmol) at 80% yield. A small amount of the (3*R*)-diastereomer (160 mg, 0.27 mmol) was also isolated (in 9% yield, related to **2**). No starting material was recovered. **R_f_** = 0.29 in hexanes–AcOEt (2:1, *v/v*) as an eluent. **^1^H NMR** (400 MHz, CDCl_3_) δ: 5.65 (t, J = 3.7 Hz, 1H), 5.64–5.62 (m, 1H), 4.88 (t, J = 4.0 Hz, 1H), 4.44–4.41 (m, 1H), 4.41–4.39 (m, 1H), 3.31 (dd, J = 14.9, 3.7 Hz, 1H), 2.40–2.30 (m, 2H), 2.30–2.22 (m, 1H), 2.11 (dd, J = 14.8, 3.7 Hz, 1H), 1.93 (s, 3H), 1.89 (s, 3H), 1.64–1.55 (m, 2H), 1.46 (s, 3H), 1.37 (s, 3H), 1.33–1.20 (m, 10H), 1.12 (d, J = 7.0 Hz, 3H), 0.90 (t, J = 7.4 Hz, 3H), 0.86 (t, J = 7.2 Hz, 3H); Figure S3A. **^13^C NMR** (100 MHz, CDCl_3_) δ: 175.85, 175.78, 175.56, 170.59, 144.04, 127.08, 84.98, 84.60, 83.76, 78.60, 78.56, 77.15, 66.22, 55.13, 41.43, 38.22, 34.11, 31.61, 29.07, 28.92, 26.11, 24.87, 23.65, 22.57, 22.51, 16.16, 16.10, 14.05, 12.89, 11.62; Figure S3B. **HMQC** is displayed in Figure S3C. **HRMS-ESI**: for [C_30_H_46_O_11_ + Na]^+^ calculated *m/z*: 605.29323 Da, found *m/z*: 605.29364; Figure S3D.

#### 2.2.4. Preparation of 2,4,6-Trichlorobenzoic (*Z*)-2-Methylbut-2-Enoic Anhydride

To a stirred solution of angelic acid (1.0 g, 10 mmol) and TEA (1.4 mL, 10 mmol) in dry toluene (30 mL) under an argon atmosphere, 2,4,6-trichloroenzoyl chloride (2.93 g, 12 mmol) was added dropwise via a syringe at 20 °C. The reaction was stirred 20 h at 20 °C; the temperature was then increased to 55 °C, and the stirring was continued for 1 h. After cooling to RT (20 °C), the formed triethylammonium chloride salt was removed by filtration through a glass filter. The solvents were removed under reduced pressure, and the residue was dried in vacuo. NMR analyses of the product showed that highly pure anhydride was obtained, and no additional purification was needed. The mixed anhydride was obtained as white crystals (3.01 g, 9.8 mmol) at 98% yield. **^1^H NMR** (400 MHz, CDCl_3_) δ: 7.36 (s, 2H), 6.28 (qq, J = 7.2, 1.5 Hz, 1H), 2.03 (dq, J = 7.2, 1.5 Hz, 3H), 1.96–1.88 (m, 3H); Figure S8A. **^13^C NMR** (100 MHz, CDCl_3_) δ: 163.28, 157.53, 143.20, 137.62, 133.06 (2C), 129.78, 128.33 (2C), 126.89, 20.30, 16.07; Figure S8B. **HMQC** is displayed in Figure S8C [28].

#### 2.2.5. Synthesis of 8-*O*-(2-(*S*)-Methylbutanoyl)-8-*O*-Debutanoyl Thapsigargin (**4**)

To a partly dissolved α-hydroxy ester **3** (500 mg, 0.86 mmol) in anhydrous toluene (1 mL) was added NaHCO_3_ (289 mg, 3.4 mmol) and 2,4,6-trichlorobenzoic (*Z*)-2-methylbut-2-enoic anhydride (524 mg, 1.72 mmol; dissolved in toluene, 2 mL) under an argon atmosphere. A gentle stream of argon was blown over the solution to slowly evaporate the toluene while reaction mixture was stirred for 18 h at 90 °C. After cooling to room temperature, the mixture was diluted with AcOEt (50 mL). The organic phase was washed with brine (2 × 50 mL). The organic layer was dried over Na_2_SO_4_, filtered, and concentrated under reduced pressure. The residue was chromatographed over a silica gel column (length of 15 cm, a diameter of 4 cm; hexanes–AcOEt, (2:1, *v/v*) as an eluent) to give angelate ester **4** (460 mg, 0.69 mmol) as a yellow powder at 80% yield. Unreacted starting hydroxyester **3** (45 mg, 77 μmol) was recovered. **R_f_** = 0.43 using hexanes–AcOEt (2:1, *v/v*) as an eluent. **^1^H NMR** (400 MHz, CDCl_3_) δ: 6.10 (qq, J = 7.2, 1.5 Hz, 1H), 5.68 (m, 1H), 5.65–5.63 (m, 1H), 5.63–5.61 (s, 1H), 5.47 (t, J = 3.8 Hz, 1H), 4.33 (s, 1H), 3.09 (dd, J = 14.7, 3.7 Hz, 1H), 2.36–2.28 (m, 2H), 2.28–2.25 (m, 1H), 2.22 (dd, J = 14.8, 3.8 Hz, 1H), 1.99 (dq, J = 7.2, 1.5 Hz, 3H), 1.91 (m, 3H), 1.88 (s, 3H), 1.86 (s, 3H), 1.64–1.54 (m, 2H), 1.48 (s, 3H), 1.39 (s, 3H), 1.35–1.22 (m, 10H), 1.14 (d, J = 7.2 Hz, 3H), 0.90 (t, J = 7.4 Hz, 3H), 0.88 (t, J = 7.0 Hz, 3H); Figure S4A. **^13^C NMR** (100 MHz, CDCl_3_) δ: 175.55, 175.48, 172.63, 170.96, 167.09, 141.74, 138.74, 130.27, 127.42, 84.63, 84.15, 78.58, 78.56, 77.71, 76.85, 66.19, 57.30, 41.42, 38.29, 34.20, 31.66, 29.05, 28.98, 26.14, 24.80, 23.17, 22.59, 22.57, 20.58, 16.28, 16.17, 15.81, 14.07, 12.96, 11.63; Figure S4B. **HMQC** is displayed in Figure S4C. **HRMS-ESI**: for [C_35_H_52_O_12_ + Na]^+^ calculated *m/z*: 687.33510 Da, found *m/z*: 687.33542; Figure S4D.

#### 2.2.6. Synthesis of 8-*O*-Debutanoyl Thapsigargin (**5**)

To a solution of **4** (1.65 g, 2.48 mmol) in MeOH (100 mL), cooled to 0 °C, TEA (6.9 mL, 68.3 mmol) was added via a syringe and the mixture was left to stir at 20 °C for 21 h. Then, pH was adjusted by adding a solution of KHSO_4_ (10%, 70 mL) until it was slightly acidic (5–6 pH). The solvent (MeOH) was slowly evaporated, and the residue was dissolved in AcOEt (100 mL). The organic phase was washed with brine (2 × 100 mL) and dried over Na_2_SO_4_, filtered and the solvents were evaporated. Chromatography of the residue was performed over a silica gel column (length of 30 cm, a diameter of 4 cm; using hexanes–AcOEt (1:1, *v/v*) as an eluent) yielded triol **5** (1.16 g, 2.0 mmol) as a white powder at 80% yield. Unreacted starting methyl butanoate **4** (298 mg, 0.44 mmol) was recovered. **R_f_** = 0.44 using hexanes–AcOEt (1:1, *v/v*) as an eluent. **^1^H NMR** (400 MHz, CDCl_3_) δ: 6.11 (qq, J = 7.2, 1.5 Hz, 1H), 5.82–5.77 (s, 1H), 5.68 (m, 1H), 5.44 (t, J = 3.8 Hz, 1H), 4.37–4.32 (m, 1H), 4.23–4.18 (m, 1H), 2.83 (dd, J = 14.8, 3.6 Hz, 1H), 2.46 (dd, J = 14.6, 3.5 Hz, 1H), 2.34–2.22 (m, 2H), 1.98 (dq, J = 7.1, 1.5 Hz, 3H), 1.91–1.90 (m, 3H), 1.90 (s, 3H), 1.84 (s, 3H), 1.64–1.55 (m, 2H), 1.49 (s, 3H), 1.43 (s, 3H), 1.33–1.22 (m, 8H), 0.90–0.83 (m, 3H); Figure S5A. **^13^C NMR** (100 MHz, CDCl_3_) δ: 175.63, 172.65, 171.33, 167.29, 140.92, 138.90, 130.08, 127.39, 85.39, 84.09, 79.58, 79.35, 77.98, 77.21, 68.61, 57.43, 39.08, 34.26, 31.66, 29.07, 28.96, 24.78, 22.80, 22.70, 22.59, 20.56, 16.24, 15.83, 14.07, 12.85; Figure S5B. **HMQC** is displayed in Figure S5C. **HRMS-ESI**: for [C_30_H_44_O_11_ + Na]^+^ calculated *m/z*: 603.27805 Da, found *m/z*: 603.27758; Figure S5D. The spectra were identical to those previously described [29].

#### 2.2.7. Preparation of Boc-*N*-12-Aminododecanoic Acid

To a suspension of 12-aminododecanoic acid (5.0 g, 23.3 mmol) and TEA (3.9 mL, 28 mmol) in MeOH (75 mL), Boc_2_O (5.05 g, 23.3 mmol) was added, and the temperature increased to 60 °C. The mixture was left to stir for 16 h. After cooling to room temperature, the solvents were evaporated. The residue was dissolved in AcOEt (100 mL), and the mixture was washed with diluted HCl solution (0.25 M, 2 × 40 mL) and brine (100 mL). Subsequently, the separated organic layer was dried over Na_2_SO_4_, filtered, and the solvent was evaporated. The NMR spectra of the product revealed that no further purification was needed. The acylation precursor Boc-*N*-12-aminododecanoic acid (7.1 g, 22.5 mmol) was obtained as a white solid at 97% yield (in [27], the yield was 93%). **^1^H NMR** (400 MHz, CDCl_3_) δ: 3.15–2.99 (m, 2H), 2.33 (t, J = 7.4 Hz, 2H), 1.62 (p, J = 7.1 Hz, 2H), 1.44 (s, 9H), 1.37–1.12 (m, 16H); Figure S9A. **HRMS-ESI**: for [C_17_H_33_NO_4_ + Na]^+^ calculated *m/z*: 338.23018 Da, found *m/z*: 338.23052; Figure S9B. The spectra were identical to those previously described [30].

#### 2.2.8. Synthesis of 8-*O*-(Boc-*N*-12-Aminododecanoyl)-8-*O*-Debutanoyl Thapsigargin (**6**)

To a solution of triol **5** (880 mg, 1.52 mmol) in anhydrous DCM (40 mL) under an argon atmosphere was added Boc-*N*-12-aminododecanoic acid (716 mg, 2.28 mmol), EDC (470 mg; 3.03 mmol), and 4-DMAP (92 mg, 0.75 mmol). The reaction was left to stir for 15 h at 20 °C. Subsequently, the solvent was evaporated and the residue was redissolved in AcOEt (100 mL). The mixture was washed with brine (2 × 100 mL). The organic phase was dried over Na_2_SO_4_, filtered, and the solvents were evaporated. Chromatography of the residue over a silica gel column (length of 30 cm, diameter of 4 cm; using hexanes–AcOEt (2:1, *v/v*) as an eluent) afforded **6** (**Boc-12ADT**; 1.27 g, 1.45 mmol) as a white powder at 95% yield. **R_f_** = 0.52 using hexanes–AcOEt (2:1, *v/v*) as an eluent. **^1^H NMR** (400 MHz, CDCl_3_) δ: 6.10 (qq, J = 7.2, 1.5 Hz, 1H), 5.68 (s, 1H), 5.67–5.64 (m, 1H), 5.63 (t, J = 3.7 Hz, 1H), 5.48 (t, J = 3.9 Hz, 1H), 4.24 (m, 1H), 3.08 (t, J = 7.2 Hz, 2H), 2.97 (dd, J = 15.0, 3.6 Hz, 1H), 2.34 (dd, J = 14.6, 3.5 Hz, 1H), 2.32–2.28 (m, 2H), 2.28–2.24 (m, 2H), 1.99 (dq, J = 7.2, 1.6 Hz, 3H), 1.91 (m, 3H), 1.89 (s, 3H), 1.86 (s, 3H), 1.64–1.59 (m, 2H), 1.59–1.54 (m, 2H), 1.48 (s, 3H), 1.46–1.44 (m, 2H), 1.43 (s, 9H), 1.40 (s, 3H), 1.33–1.23 (m, 22H), 0.86 (t, J = 7.1 Hz, 3H); Figure S6A. **^13^C NMR** (100 MHz, CDCl_3_) δ: δ 175.37, 172.66, 172.56, 170.59, 167.09, 156.83, 141.69, 138.69, 130.20, 127.44, 84.47, 84.11, 78.59 (2 C), 77.76, 77.21, 76.80, 66.11, 57.71, 41.25, 38.29, 34.57, 34.24, 31.66, 29.97, 29.32, 29.21, 29.15, 29.09, 29.06, 29.04, 28.97, 28.94, 28.42 (3 C), 26.63, 24.81, 24.34, 22.72, 22.59, 22.56, 20.57, 16.19, 15.80, 14.07, 12.96; Figure S6B. **HMQC** is displayed in Figure S6C. **HRMS-ESI**: for [C_47_H_75_NO_14_ + Na]^+^ calculated *m/z*: 900.50798 Da, found *m/z*: 900.50827; Figure S6D. The spectra were identical to those previously obtained [29].

#### 2.2.9. Deprotection of Boc group to afford 8-*O*-(12-aminododecanoyl)-8-*O*-debutanoyl thapsigargin (**7**)

To a solution of product **6** (93 mg, 0.11 mmol) in DCM (5 mL) was added TFA (5 mL) and 3 drops of distilled H_2_O. The reaction was stirred for 90 min at 20 °C. The mixture was carefully evaporated with an addition of toluene (10 mL). The product was further purified by silica gel column chromatography (length of 5 cm, a diameter of 4 cm; using DCM–MeOH (10:1, *v/v*) as an eluent) to give **7** as a colorless amorphous solid (**12ADT**; 80 mg, 0.1 mmol) at 94% yield. Note that no bocylated started material was recovered. **R_f_** = 0.36 using DCM–MeOH (6:1, *v/v*) as an eluent). **^1^H NMR** (400 MHz, DCl_3_) δ ppm: 6.16 (qq, J = 7.2, 1.5 Hz, 1H), 5.70–5.66 (s, 1H), 5.65 (m, 1H), 5.57 (t, J = 3.7 Hz, 1H), 5.49 (t, J = 3.9 Hz, 1H), 4.36–4.31 (m, 1H), 3.68–3.62 (m, 1H), 2.96 (dd, J = 14.6, 3.7 Hz, 1H), 2.93–2.87 (m, 1H), 2.38–2.32 (m, 2H), 2.32–2.30 (m, 1H), 2.30–2.25 (m, 2H), 1.98 (dq, J = 7.2, 1.6 Hz, 3H), 1.93–1.89 (m, 3H), 1.86 (s, 3H), 1.84 (s, 3H), 1.78–1.70 (m, 2H), 1.67–1.61 (m, 2H), 1.61–1.54 (m, 2H), 1.39 (s, 3H), 1.34 (s, 3H), 1.37–1.26 (m, 20H), 0.92–0.86 (m, 3H); Figure S7A. **^13^C NMR** (100 MHz, CDCl_3_) δ ppm: 176.68, 172.86, 172.28, 170.37, 167.12, 139.58, 138.10, 131.94, 127.38, 84.47, 84.26, 78.04, 77.99, 77.88, 76.57, 65.97, 57.62, 47.43, 39.33, 37.97, 34.09, 33.74, 31.44, 29.17, 29.10, 29.08, 28.98, 28.80, 28.70, 28.66, 27.16, 26.27, 24.55, 24.15, 22.25, 21.92, 21.26, 19.33, 14.65, 14.41, 13.02, 11.55.; Figure S7B. **HMQC** is displayed in Figure S7C. **HRMS-ESI**: for [C_42_H_68_NO_12_ + H]^+^ calculated *m/z*: 778.47360 Da, found *m/z*: 778.47428; Figure S7D. The spectra were identical to those previously described [29].

## 3. Results and Discussion

The goal of this work is to develop a procedure for the large-scale production of **12ADT** (**7**). A synthesis of **Tg** containing relatively few steps, starting with **nTb** on a milligram scale, has been published. However, gram-scale synthesis of **12ADT** (**7**) has not yet been reported. Decagram amounts of **Tb** from a kilogram of fruit extract of *L. trilobum* (Figure 2) can be obtained in a sustainable way. The structural similarities between **Tb** and **nTb** make this compound an obvious starting point for the preparation of **12ADT** (**7**) on a gram scale. Isolation and production technologies of pharmacologically active sesquiterpene lactones [31] were recently reviewed [32,33]. A protocol for isolation of crystalline **Tb** on a decagram scale by supercritical CO_2_ extraction, followed by flash chromatography, is described in Section 2.1.

**Scheme 1:**
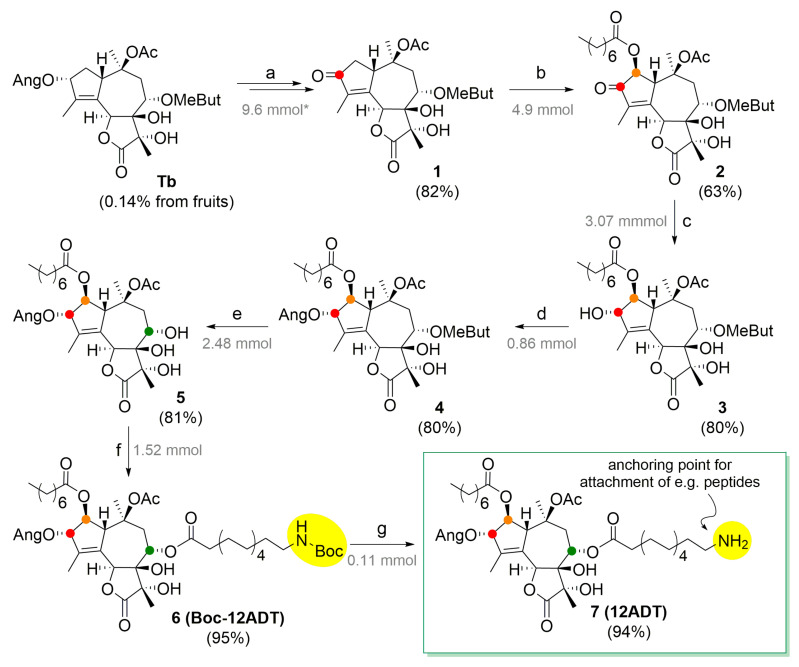
Transformation of **Tb** to a **Tg** analog **12ADT** (**7**). Reagents and conditions: (**a**) CrO_3_, HF, H_2_O, MeCN, 95 °C, 2.5 h; (**b**) octanoic acid, Mn(OAc)_3_ · 2H_2_O, 120 °C, 6 h; (**c**) Zn(BH_4_)_2_, Et_2_O, −20 °C, 3.5 h, followed by H_2_O; (**d**) 2,4,6-trichlorobenzoic (*Z*)-2-methylbut-2-enoic anhydride, NaHCO_3_, toluene, argon atmosphere, 90 °C, 18 h; (**e**) TEA, MeOH, 0→20 °C, 21 h, followed by KHSO_4_; (**f**) boc-12-aminododecanoic acid, EDC, 4-DMAP, DCM, argon atmosphere, 20 °C, 15 h; (**g**) TFA, DCM, H_2_O, 20 °C, 90 min. * Under the arrows, the scale of the starting material is specified in grey.

A procedure for transformation of **Tb** to **12ADT** (**7**) in 7 steps, with an overall yield of 24% (Scheme 1), is described. An overview of the reactions, conditions, molar scale, and the results obtained in the present work is given in Scheme S1 in comparison with previously developed protocols for milligram-scale transformation of **nTb** to **Tg**.

The key step of **Tb** transformation to **12ADT** (**7**) is the insertion of an octanoyl moiety to the C-2 position of the **Tb** skeleton (Figure 1A). This was achieved via ketone **1**, in which H-2 is activated [26,27] (Scheme 1a). This reaction path involves the selective removal of the angeloyl moiety (Ang) from *O*-3 and subsequent oxidation of the formed alcohol. A similar reaction was performed with **nTb** [26,27]. In situ oxidation of the intermediate alcohol prevents degradation of labile 3-*O*-desangelyl-**Tb**. This optimized method was scaled up, enabling the synthesis of ketone **1** in gram amounts (Section 2.2.1). Ketone **1** (Scheme 1a) was obtained after processing and chromatographic purification at 82% yield.

Ketone **1** was converted into **2** [27] using Mn(OAc)_3_ · 2H_2_O and octanoic acid as reagents in benzene. The developed procedure resulted in optimal reaction conditions to get ketoester **2** at 63% yield after upscaling [26,27]. The reaction proceeded smoothly, and a procedure for removing the formed sodium octanoate was developed (Section 2.2.2). Recovery of ketone **1** increased the yield to 85% yield of α-ketoester **2**. A likely reaction mechanism explaining both regio- and stereoselective formation of the product is shown in Scheme S2.

For reintroduction of the angelate ester to the *O*-3 position, C-3 ketone **2** has to be stereoselectively reduced to alcohol **3** (Scheme 1c). The procedure developed by Crestey et al. [26] (Scheme S1c) was followed. The reduction gave alcohol **3** at 80% yield (Section 2.2.3). That is a slightly lower yield than the previously reported 87% of alcohol **s3a** obtained from ketone **s2a** (Scheme S1c) [27].

Esterification with angelic acid is complicated by the easy isomerization of angelic esters into tiglic esters [34]. 3-OH angeloylation succeeded using 2,4,6-trichlorobenzoic (*Z*)-2-methylbut-2-enoic anhydride (Section 2.2.4) as a reagent [28] in a toluene solution. A too-diluted solution afforded a poor yield, whereas a too-concentrated solution resulted in problems with stirring. By blowing argon over the solution overnight, a melt-like solution appeared, resulting in the maximal conversion of **3** to product **4**. Due to these specific conditions (Section 2.2.5), optimal yield (80%) was obtained by running a number of small-scale reactions in parallel. This yield was a significant enhancement in comparison with previous reports [26,27] (see Scheme S1d). The use of the purified mixed anhydride instead of in situ generated anhydride is likely to be the key factor for improving the yield [26,27].

The removal of the *O*-8 ester group from compound **4** (2.48 mmol) was performed by a validated mild transesterification method (Scheme S1e) using the MeOH-TEA solvent system [35,36]. A precautionary measure of adjusting the pH to being slightly acidic (5–6 pH), with 10% KHSO_4_ before the evaporation of the solvent under reduced pressure, was taken, and the reaction (Section 2.2.6) afforded **5** at 80% yield. The starting material **4** (0.44 mmol) was partially recovered, and, thus, the recalculated yield reached 98%.

The regioselective insertion of previously prepared (Section 2.2.7) Boc-*N*-12-aminododecanoyl moiety to 8-OH of triol **5** was performed by Steglich esterification using EDC/4-DMAP (Scheme S1f) to give **Boc-12ADT** (**6**) at 95% yield (Section 2.2.8), which is an improvement compared to a previous study [30] (Scheme S1f).

Finally, the cleavage of the Boc group was performed (Section 2.2.9) according to standard procedures [18], i.e., by using a solution of TFA in DCM (Scheme 1g). The lability of the free amine group present within **12ADT** (**7**) makes us suggest that only the Boc-protected derivative **Boc-12ADT** (**6**) is stored. This reaction was only performed to show that **Boc-12ADT** (**7**) is easily transformed into the free amine, which should then be coupled with the chosen targeting vector right away.

## 4. Conclusions

This study describes a successfully developed procedure for large scale isolation of **Tb** by CO_2_ supercritical extraction from harvested fruits. A procedure for converting **Tb** to **12ADT** (**7**) on a gram scale was developed. The optimized synthetic route to **12ADT** (**7**) from **Tb** involved only seven synthetic steps, with **12ADT** (**7**) overall yield of 24%, which outperforms analogous previously published methods. The presented work offers a procedure for making **Tg** and its derivatives more accessible in a sustainable way. That facilitates the preparation of **Tg** prodrugs for treatment of slowly developing cancer diseases, like hepatocellular carcinoma and prostate cancer, and eases their clinical evaluation process.

## Supplementary Materials

The following are available online at https://www.mdpi.com/2218-273X/10/12/1640/s1. Scheme S1: The reactions adopted from the literature. Scheme S2: Proposed mechanism of α-oxyacylation. Supplementary Figures S1A–S9B document the analytical identification (*NMR and HRMS*), with ^1^H and ^13^C NMR assignments (Section 2.2).

## References

[1] Doan N.T.Q., Paulsen E.S., Sehgal P., Moeller J.V., Nissen P., Denmeade S.R., Isaacs J.T., Dionne C.A., Christensen S.B. (2015). Targeting thapsigargin towards tumors. Steroids.

[2] Ma Z., Fan C., Yang Y., Di S., Hu W., Li T., Zhu Y., Han J., Xin Z., Wu G. (2016). Thapsigargin sensitizes human esophageal cancer to TRAIL induced apoptosis via AMPK activation. Sci. Rep..

[3] Huang F., Wang P., Wang X. (2018). Thapsigargin induces apoptosis of prostate cancer through cofilin-1 and paxillin. Oncol. Lett..

[4] Wu L., Huang X., Kuang Y., Xing Z., Deng X., Luo Z. (2019). Thapsigargin induces apoptosis in adrenocortical carcinoma by activating endoplasmic reticulum stress and the JNK signaling pathway: An in vitro and in vivo study. Drug Des. Devel. Ther..

[5] Denmeade S.R., Mhaka A.M., Rosen D.M., Brennen W.N., Dalrymple S., Dach I., Olesen C., Gurel B., DeMarzo A.M., Wilding G. (2012). Engineering a prostate specific membrane antigen activated tumor endothelial cell prodrug for cancer therapy. Sci. Transl. Med..

[6] Škubník J., Jurášek M., Ruml T., Rimpelová S. (2020). Mitotic poisons in research and medicine. Molecules.

[7] Chan K.S., Koh C.G., Li H.Y. (2012). Mitosis targeted anti-cancer therapies: Where they stand. Cell Death Discov..

[8] Denmeade S.R., Jakobsen C.M., Janssen S., Khan S.R., Garrett E.S., Lilja H., Christensen S.B., Isaacs J.T. (2003). Prostate specific antigen activated thapsigargin prodrug as targeted therapy for prostate cancer. J. Natl. Cancer Inst..

[9] Peterková L., Kmoníčková E., Ruml T., Rimpelová S. (2020). Sarco/endoplasmic reticulum calcium ATPase inhibitors: Beyond anticancer perspective. J. Med. Chem..

[10] Sohoel H., Lund J.A.-M., Moller J.V., Nissen P., Denmeade S.R., Isaacs J.T., Olsen C.E., Christensen S.B. (2006). Natural products as starting materials for development of second-generation SERCA inhibitors targeted towards prostate cancer cells. Bioorg. Med. Chem..

[11] Sehgal P., Szalai P., Olesen C., Praetorius H.A., Nissen P., Christensen S.B., Engedal N., Møller J.V. (2017). Inhibition of the sarco/endoplasmic reticulum (ER) Ca(2+) ATPase by thapsigargin analogs induces cell death via ER Ca(2+) depletion and the unfolded protein response. J. Biol. Chem..

[12] Andersen T.B., Lopez C.Q., Manczak T., Martinez K., Simonsen H.T. (2015). Thapsigargin from *Thapsia,* L. to mipsagargin. Molecules.

[13] Kmoníčková E., Harmatha J., Vokáč K., Kostecká P., Farghali H., Zídek Z. (2010). Sesquiterpene lactone trilobolide activates production of interferon-γ and nitric oxide. Fitoterapia.

[14] Winther A.-M.L., Liu H., Sonntag Y., Olesen C., Le M.M., Soehoel H., Olsen C.-E., Christensen S.B., Nissen P., Moller J.V. (2010). Critical roles of hydrophobicity and orientation of side chains for inactivation of sarcoplasmic reticulum Ca^2+^ ATPase with thapsigargin and thapsigargin analogs. J. Biol. Chem..

[15] Harmatha J., Buděšínský M., Jurášek M., Zimmermann T., Drašar P., Zídek Z., Kmoníčková E., Vejvodová L. (2019). Structural modification of trilobolide for upgrading its immunobiological properties and reducing its cytotoxic action. Fitoterapia.

[16] Christensen S.B., Andersen A., Kromann H., Treiman M., Tombal B., Denmeade S., Isaacs J.T. (1999). Thapsigargin analogs for targeting programmed death of androgen independent prostate cancer cells. Bioorg. Med. Chem..

[17] Janssen S., Rosen D.M., Ricklis R.M., Dionne C.A., Lilja H., Christensen S.B., Isaacs J.T., Denmeade S.R. (2006). Pharmacokinetics, biodistribution, and antitumor efficacy of a human glandular kallikrein 2 (hK2)-activated thapsigargin prodrug. Prostate.

[18] Zimmermann T., Christensen S., Franzyk H. (2018). Preparation of enzyme-activated thapsigargin prodrugs by solid-phase synthesis. Molecules.

[19] Mahalingam D., Peguero J., Cen P., Arora S.P., Sarantopoulos J., Rowe J., Allgood V., Tubb B., Campos L. (2019). A phase II, multicenter, single-arm study of mipsagargin (G-202) as a second-line therapy following sorafenib for adult patients with progressive advanced hepatocellular carcinoma. Cancers.

[20] Mahalingam D., Wilding G., Denmeade S., Sarantopoulas J., Cosgrove D., Cetnar J., Azad N., Bruce J., Kurman M., Allgood V.E. (2016). Mipsagargin, a novel thapsigargin-based PSMA-activated prodrug: Results of a first-in-man phase I clinical trial in patients with refractory, advanced or metastatic solid tumours. Br. J. Cancer.

[21] Tarvainen I., Zimmermann T., Heinonen P., Jäntti M.H., Yli-Kauhaluoma J., Talman V., Franzyk H., Tuominen R.K., Christensen S.B. (2019). Missing selectivity of targeted 4β-phorbol prodrugs expected to be potential chemotherapeutics. ACS Med. Chem. Lett..

[22] Akinboye E.S., Rogers O.C., Isaacs J.T. (2018). 2-Fluoro-5-maleimidobenzoic acid-linked albumin drug (MAD) delivery for selective systemic targeting of metastatic prostate cancer. Prostate.

[23] Akinboye E.S., Brennen W.N., Denmeade S.R., Isaacs J.T. (2019). Albumin-linked prostate-specific antigen-activated thapsigargin and niclosamide based molecular grenades targeting the microenvironment in metastatic castration-resistant prostate cancer. Asian J. Urol..

[24] Chu H., Smith J.M., Felding J., Baran P.S. (2017). Scalable Synthesis of (−)-Thapsigargin. ACS Cent. Sci..

[25] Ley S.V., Antonello A., Balskus E.P., Booth D.T., Christensen S.B., Cleator E., Gold H., Högenauer K., Hünger U., Myers R.M. (2004). Synthesis of the thapsigargins. Proc. Natl. Acad. Sci. USA.

[26] Crestey F., Toma M., Christensen S.B. (2015). Concise synthesis of thapsigargin from nortrilobolide. Tetrahedron Lett..

[27] Doan N.T.Q., Crestey F., Olsen C.E., Christensen S.B. (2015). Chemo-and regioselective functionalization of nortrilobolide: Application for semisynthesis of the natural product 2-acetoxytrilobolide. J. Nat. Prod..

[28] Andrews S.P., Ball M., Wierschem F., Cleator E., Oliver S., Hogenauer K., Simic O., Antonello A., Hunger U., Smith M.D. (2007). Total synthesis of five thapsigargins: Guaianolide natural products exhibiting sub-nanomolar SERCA inhibition. Chemistry.

[29] Jakobsen C.M., Denmeade S.R., Isaacs J.T., Gady A., Olsen C.E., Christensen S.B. (2001). Design, synthesis, and pharmacological evaluation of thapsigargin analogues for targeting apoptosis to prostatic cancer cells. J. Med. Chem..

[30] Wang D.S., Wagner M., Butt H.J., Wu S. (2015). Supramolecular hydrogels constructed by red-light-responsive host-guest interactions for photo-controlled protein release in deep tissue. Soft Matter.

[31] Amorim M.H.R., Gil da Costa R.M., Lopes C., Bastos M.M.S.M. (2013). Sesquiterpene lactones: Adverse health effects and toxicity mechanisms. Crit. Rev. Toxicol..

[32] Kishkentayeva A.S., Adekenov S.M., Drašar P.B. (2018). Production technologies of pharmacologically active sesquiterpene lactones. Eurasian Chem. Technol. J..

[33] de Melo M.M.R., Silvestre A.J.D., Silva C.M. (2014). Supercritical fluid extraction of vegetable matrices: Applications, trends and future perspectives of a convincing green technology. J. Supercrit. Fluids.

[34] Liang X., Grue-Soerensen G., Petersen A.K., Hogberg T. (2012). Semisynthesis of ingenol 3-angelate (PEP005): Efficient stereoconservative angeloylation of alcohols. Synlett.

[35] Jurášek M., Rimpelová S., Kmoníčková E., Drašar P., Ruml T. (2014). Tailor-made fluorescent trilobolide to study its biological relevance. J. Med. Chem..

[36] Huml L., Jurášek M., Mikšátková P., Zimmermann T., Tomanová P., Buděšínský M., Rottnerová Z., Šimková M., Harmatha J., Kmoníčková E. (2017). Immunoassay for determination of trilobolide. Steroids.

