# Supplementary material for: Large Scale Conversion of Trilobolide into the Payload of Mipsagargin: 8-O-(12-Aminododecanoyl)-8-O-Debutanoylthapsigargin
Source: Biomolecules. 2020 Dec 5;10(12):1640. doi: 10.3390/biom10121640 (PMC7762042; doi:10.3390/biom10121640)
Supplement: Supplementary file 1 [file biomolecules-10-01640-s001.pdf]

Supplementary information for

Large scale conversion of trilobolide into the payload of Mipsagargin: 8-*O*-(12-aminododecanoyl)-8-*O*-debutanoylthapsigargin

Tomáš Zimmermann [1], Pavel Drašar [1], Silvie Rimpelová [2], S. Brøgger Christensen [3], Vladimir A. Khripach [4] and Michal Jurášek [1*]

[1] Department of Chemistry of Natural Compounds, University of Chemistry and Technology Prague, Technická 5, 166 28 Prague, The Czech Republic; tomas.zimmermann@vscht.cz, michal.jurasek@vscht.cz, pavel.drasar@vscht.cz
[2] Department of Biochemistry and Microbiology, University of Chemistry and Technology Prague, Technická 5, 166 28 Prague 6, The Czech Republic; silvie.rimpelova@vscht.cz
[3] Department of Drug Design and Pharmacology, University of Copenhagen; Jagtvej 162, DK-2100 Copenhagen Ø, Denmark, soren.christensen@sund.ku.dk
[4] Institute of Bioorganic Chemistry, National Academy of Sciences of Belarus, Kuprevich St. 5, 220141 Minsk, Belarus; khripach@iboch.by
* Correspondence: michal.jurasek@vscht.cz

Table of Contents:

Product 1 (TZ81) – Figures S1 2-5

Product 2 (TZ82) – Figures S2 6-9

Product 3 (TZ84) – Figures S3 10-13

Product 4 (TZ87) – Figures S4 14-17

Product 5 (TZ92) – Figures S5 18-21

Product 6 (TZ96) – Figures S6 22-25

Product 7 (TZ99) – Figures S7 26-29

2,4,6-Trichlorobenzoic (Z)-2-methylbut-2-enoic anhydride – Figures S8 30-32

Boc-*N*-12-aminododecanoic acid – Figures S9 33-34

Scheme S1 35

Scheme S2 36

Product **1** (3-oxo-3-desangeloyl trilobolide)

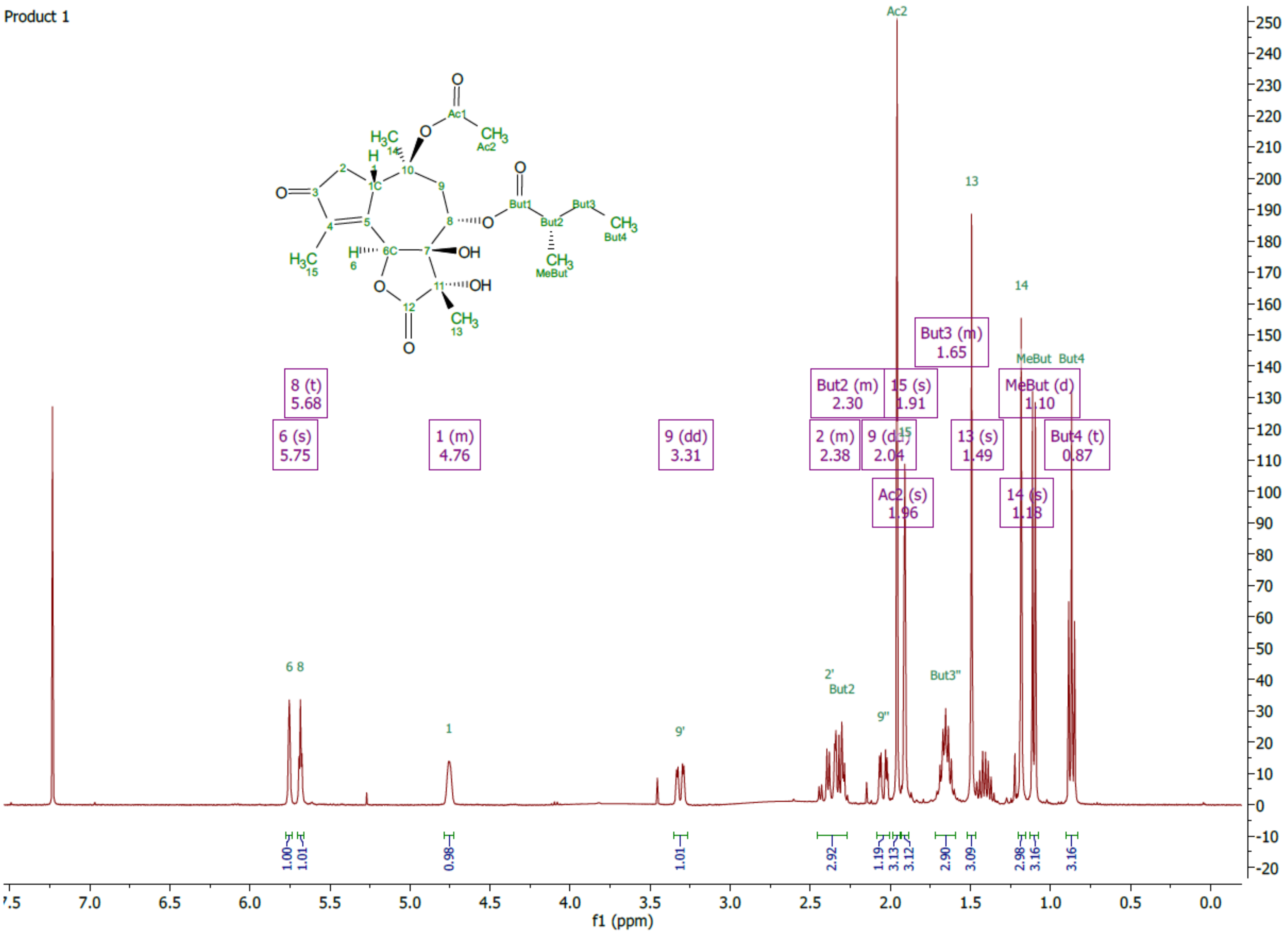

Figure S1A – ^{1}H NMR of product **1** (400 MHz, $CDCl_3$)

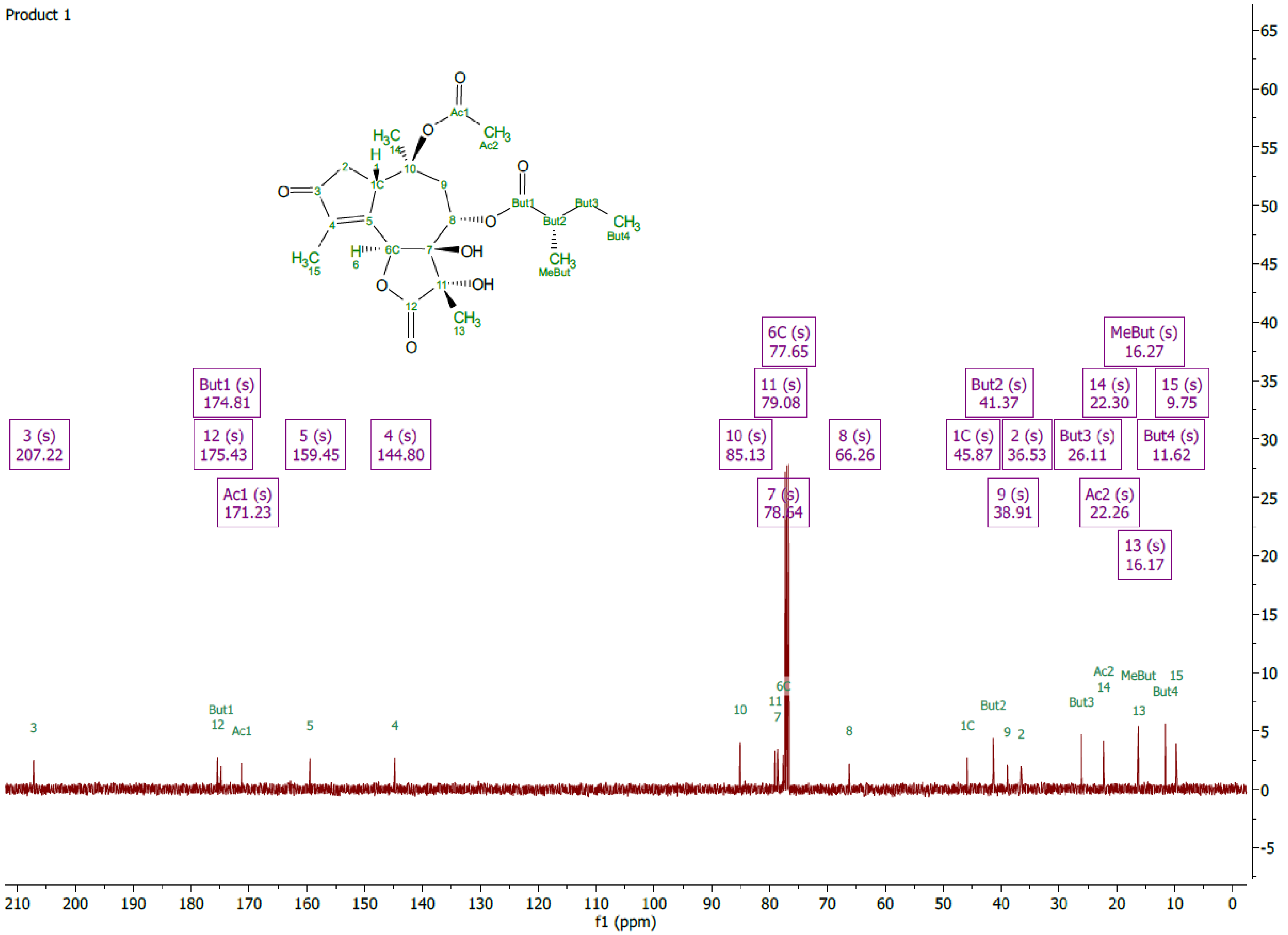

Figure S1B – ^{13}C NMR of product **1** (100 MHz, $CDCl_3$)

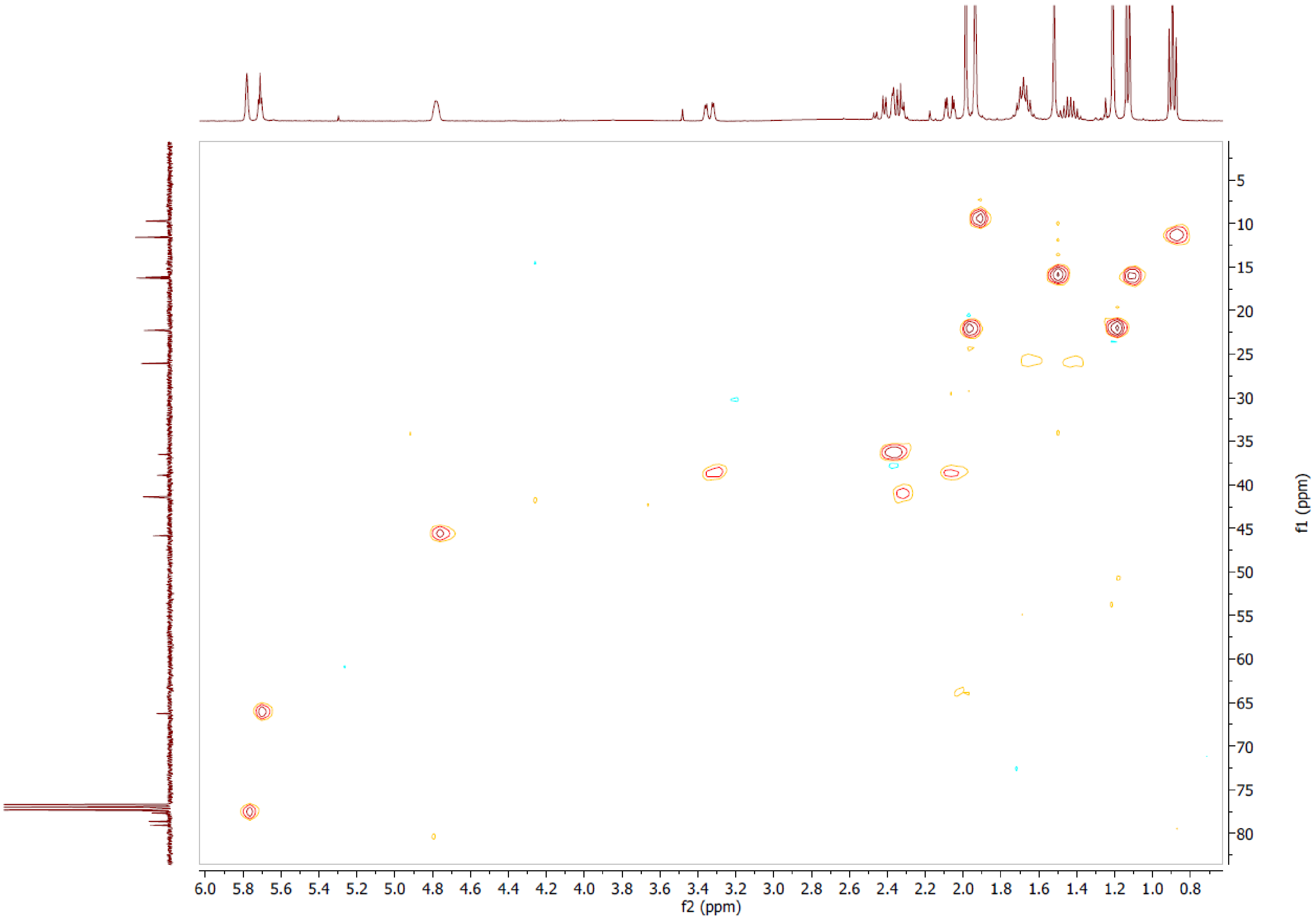

Figure S1C - HMQC of product **1**

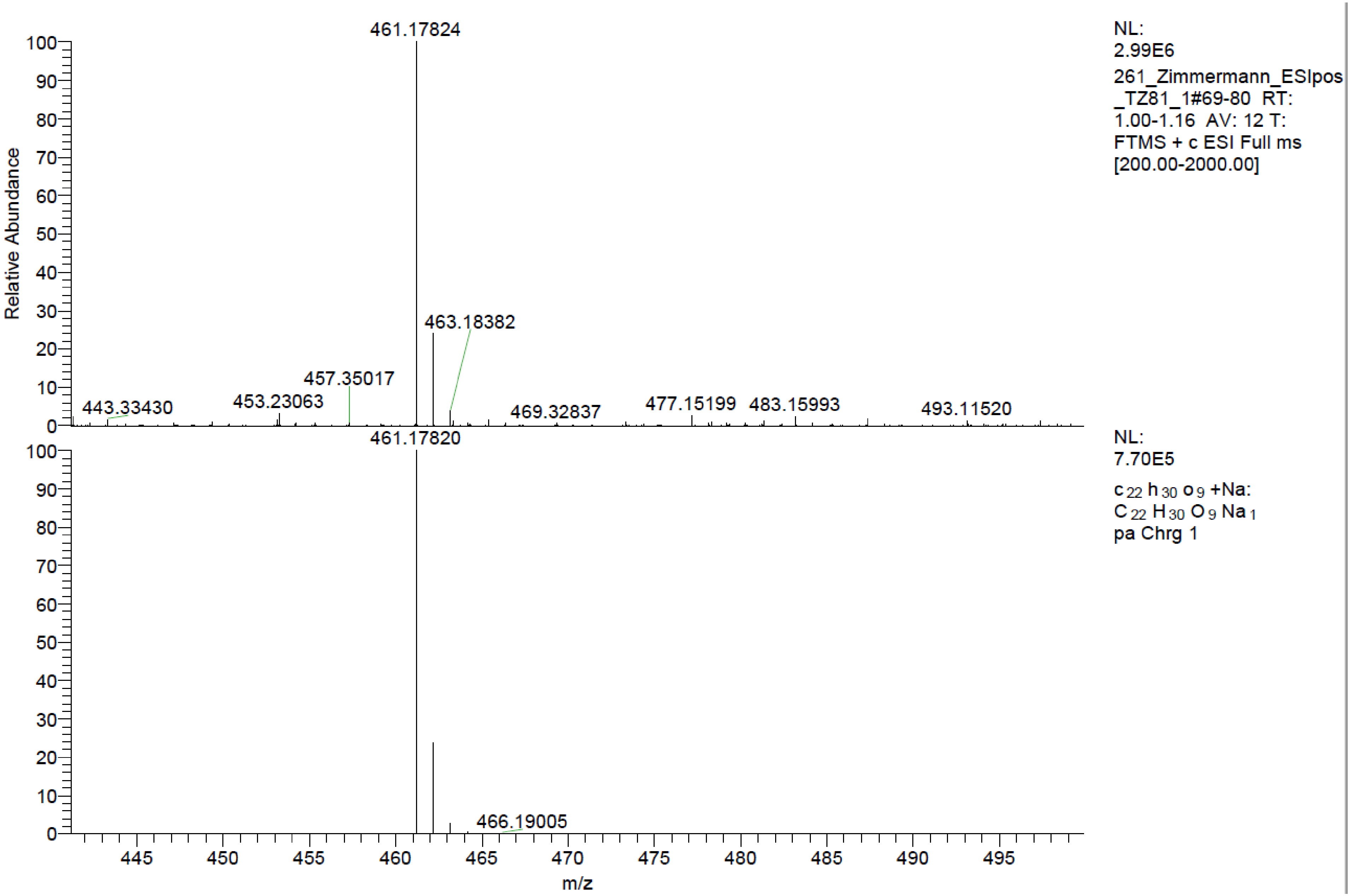

Figure S1D - HRMS of product **1** - $[C_{22}H_{30}O_9+Na]^+$ = 461.17824

Product **2** (2-octanoyl-3-oxo-3-desangeloyl trilobolide)

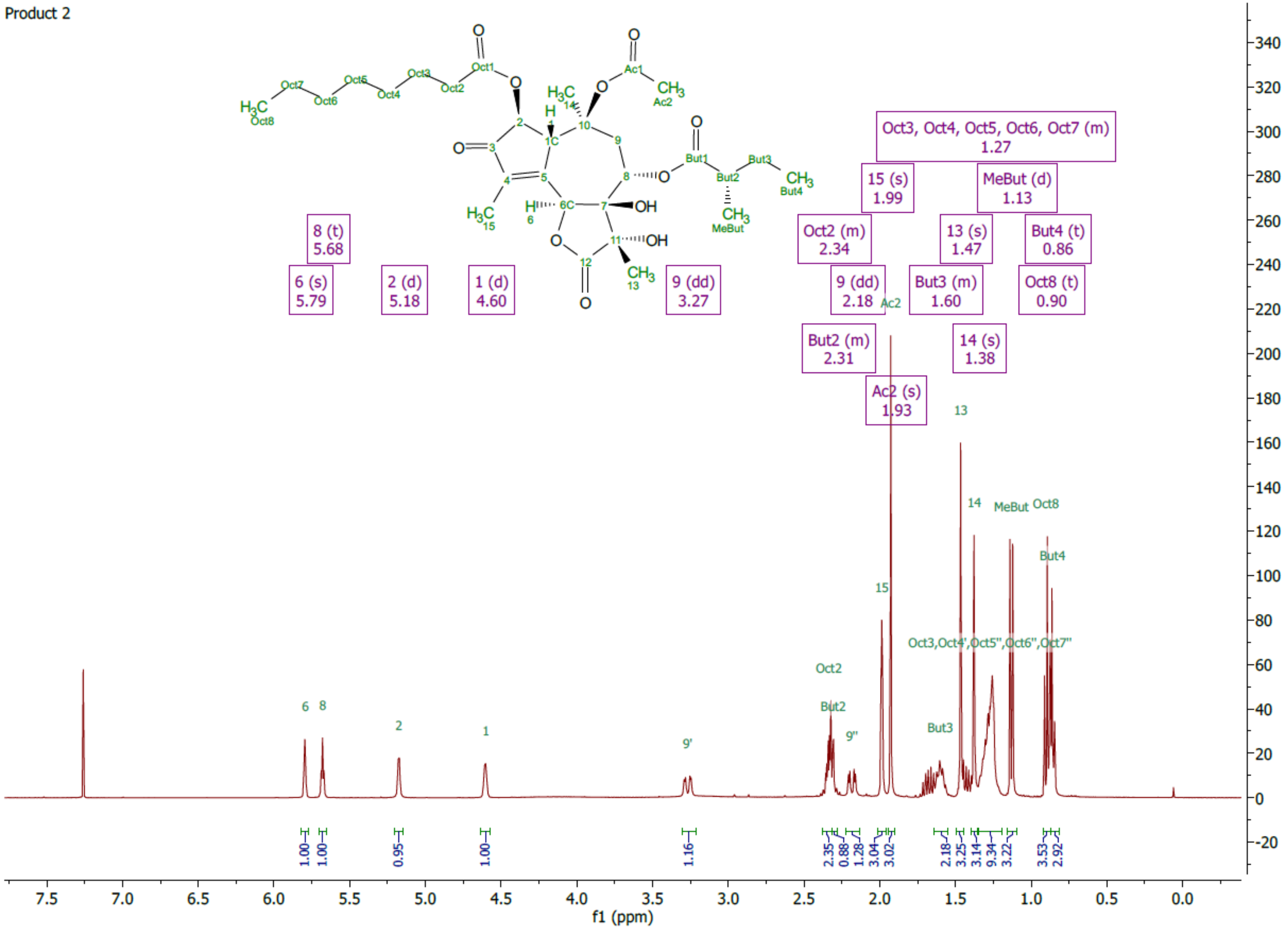

Figure S2A ^{1}H NMR of product **2** (400 MHz, $CDCl_3$)

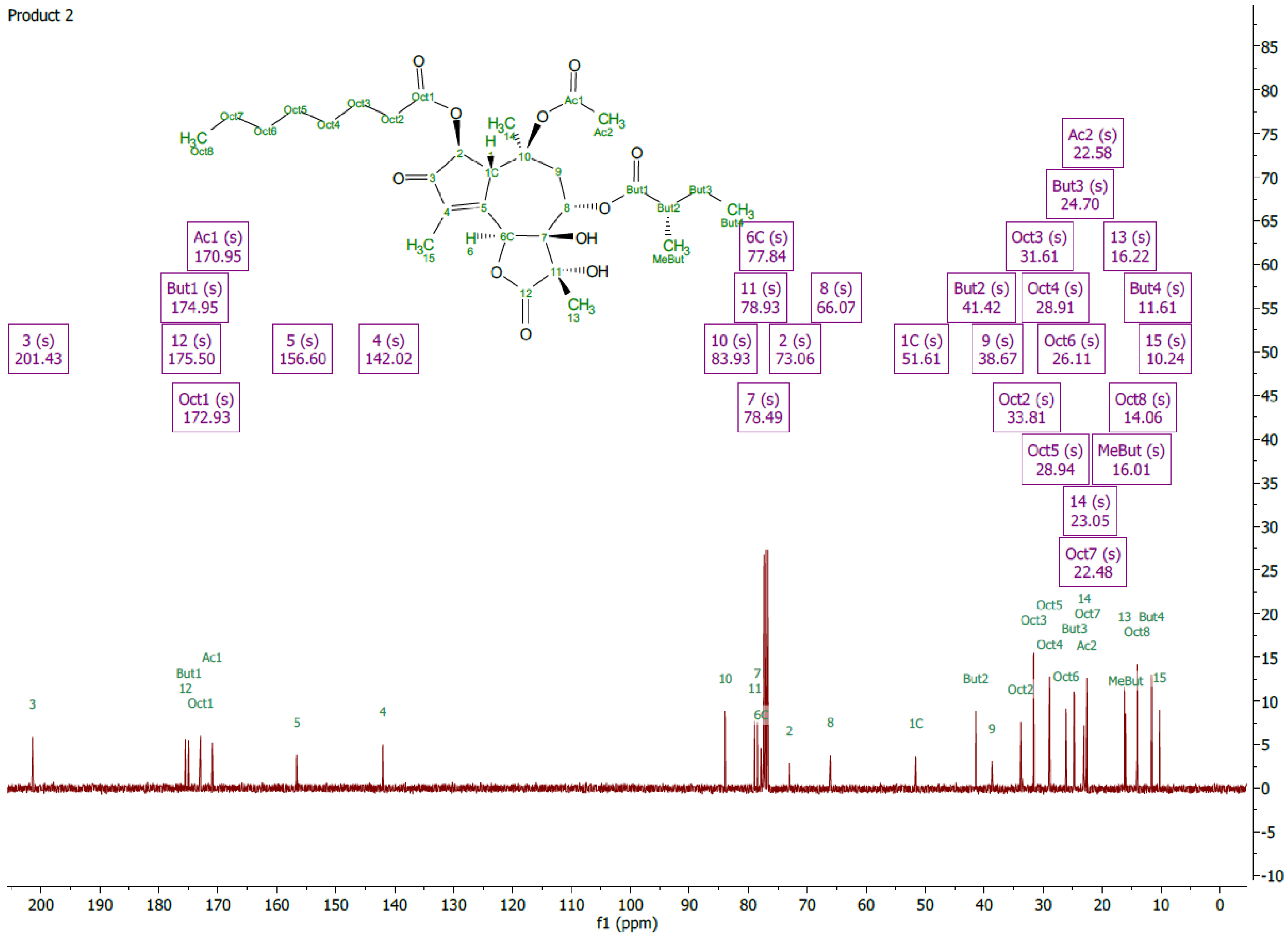

Figure S2B ^{13}C NMR of product **2** (100 MHz, $CDCl_3$)

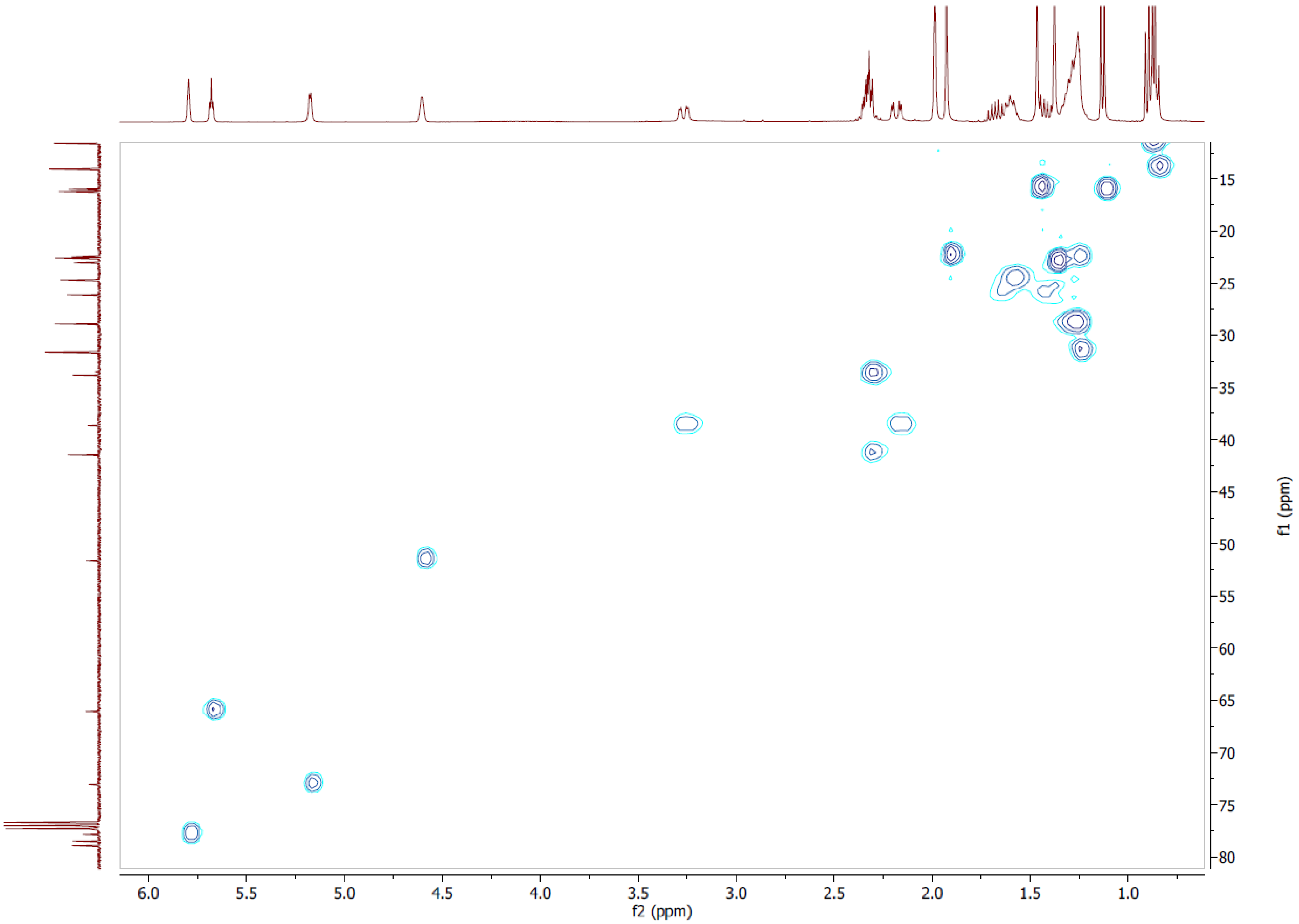

Figure S2C HMQC of product **2**

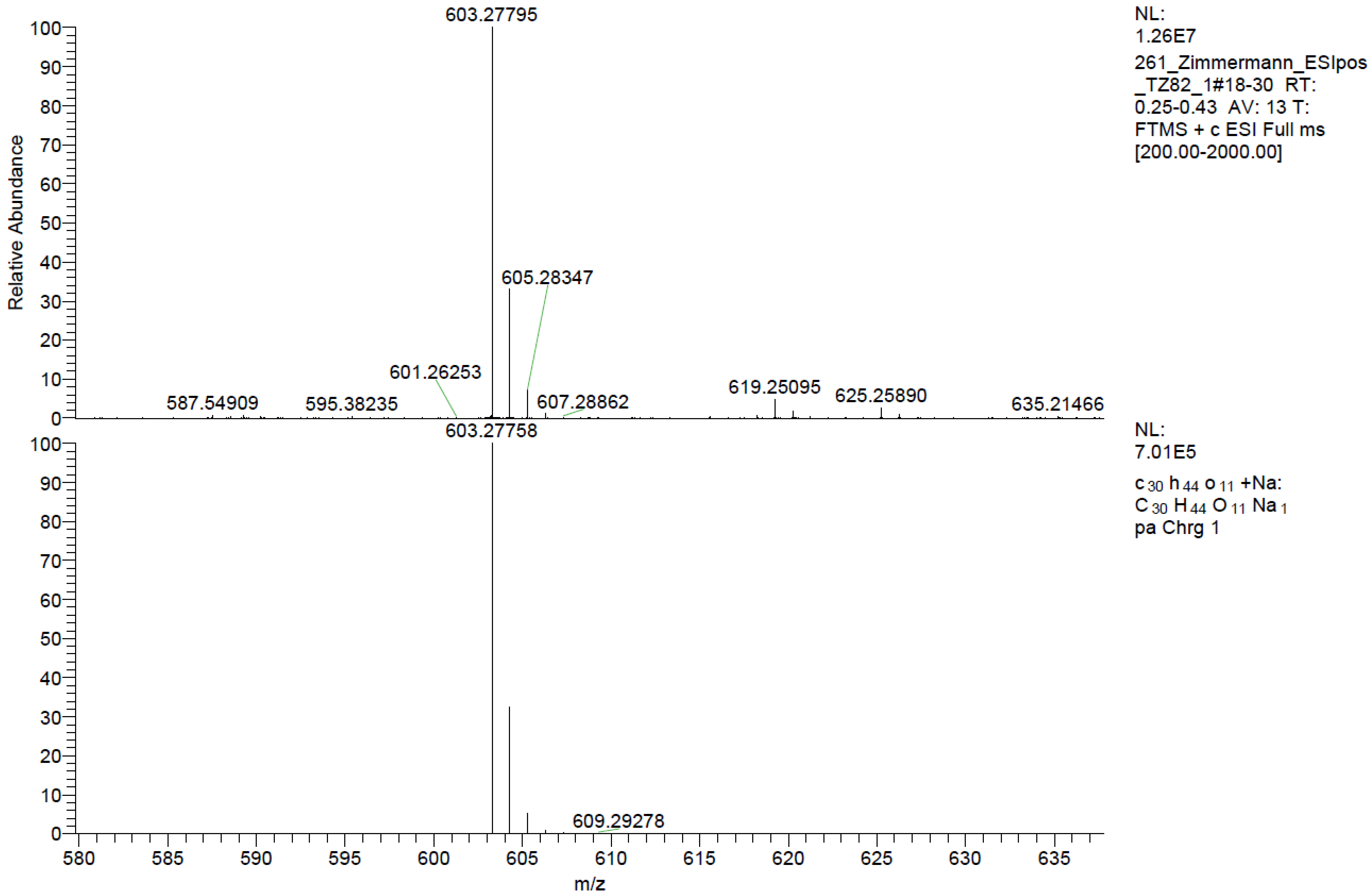

Figure S2D LCMS of product **2 -** $[C_{30}H_{44}O_{11}+Na]^+ = 603.27795$

Product **3** ((3*S*)-hydroxy-2-octanoyl-3-desangeloyl trilobolide)

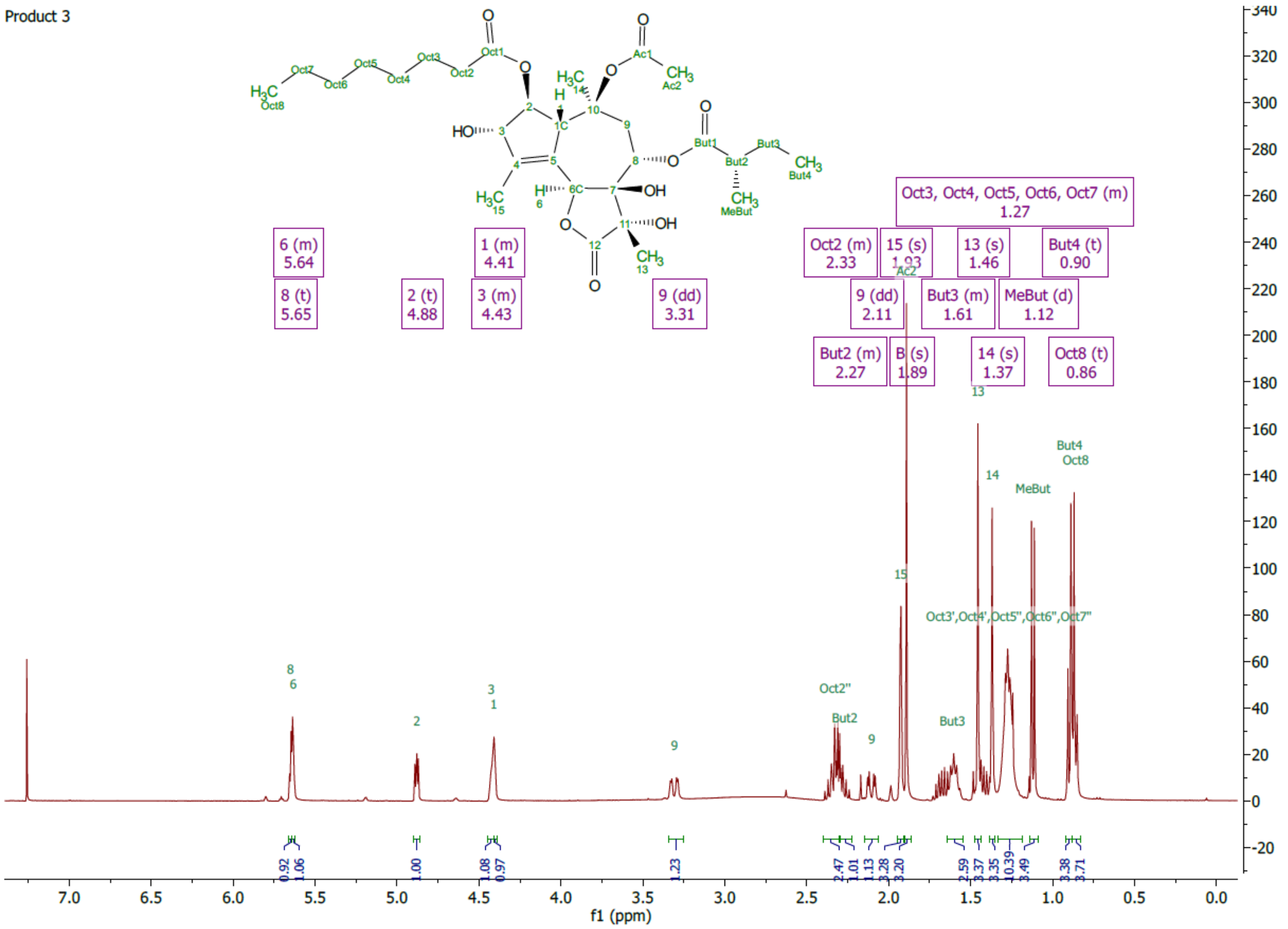

Figure S3A ^{1}H NMR of product **3** (400 MHz, $CDCl_3$)

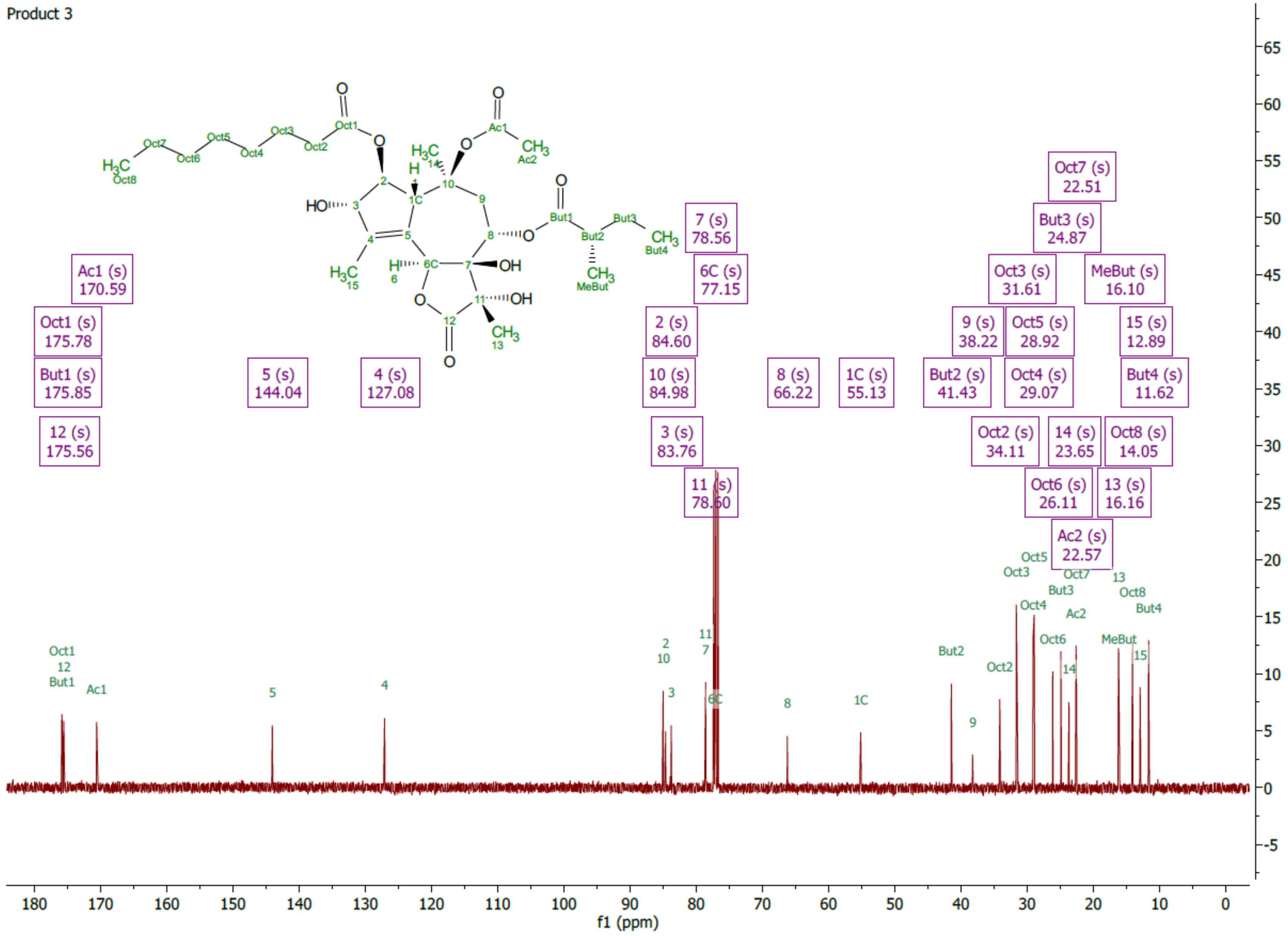

Figure S3B ^{13}C NMR of product **3** (100 MHz, $CDCl_3$)

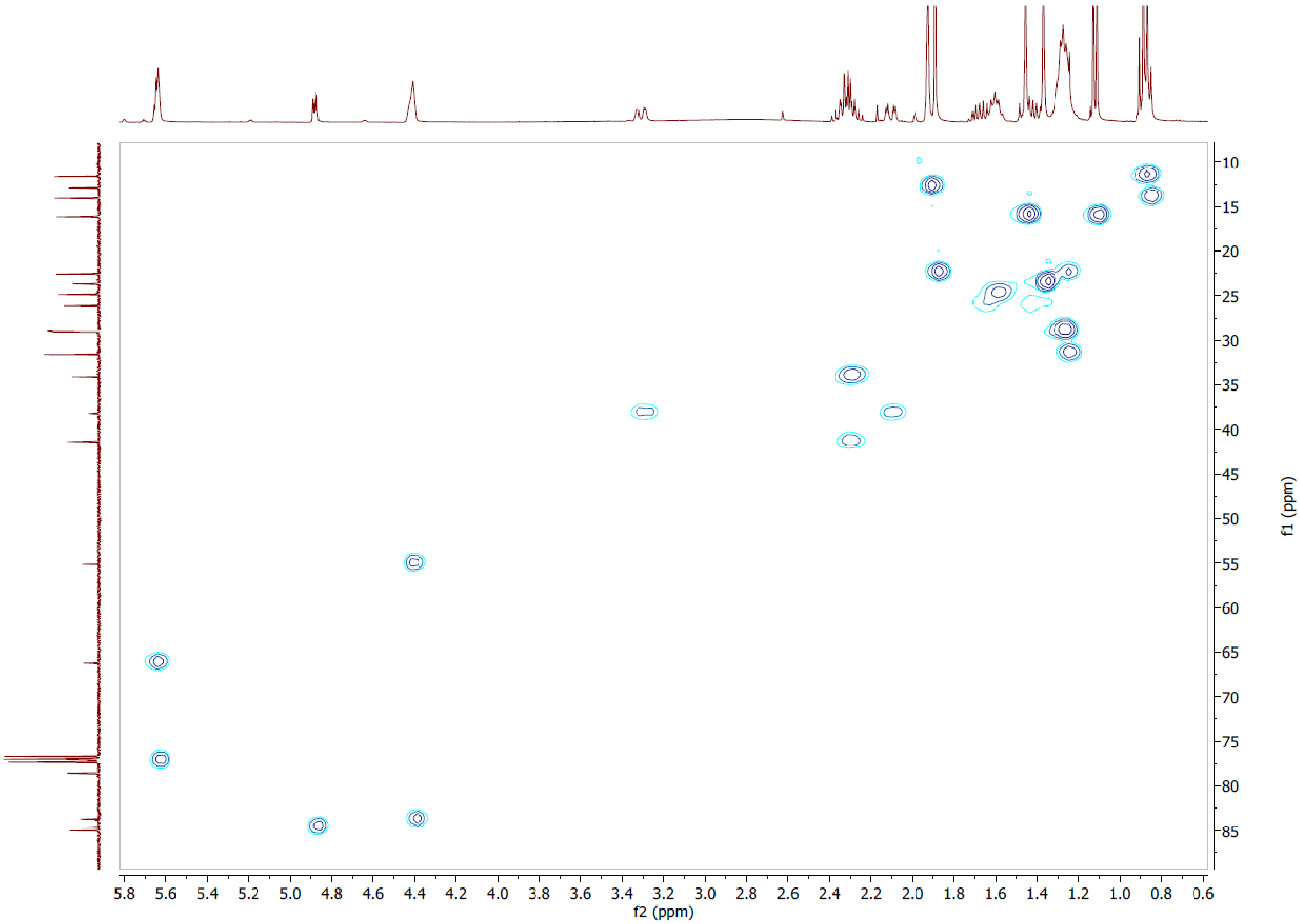

Figure S3C HMQC of product **3**

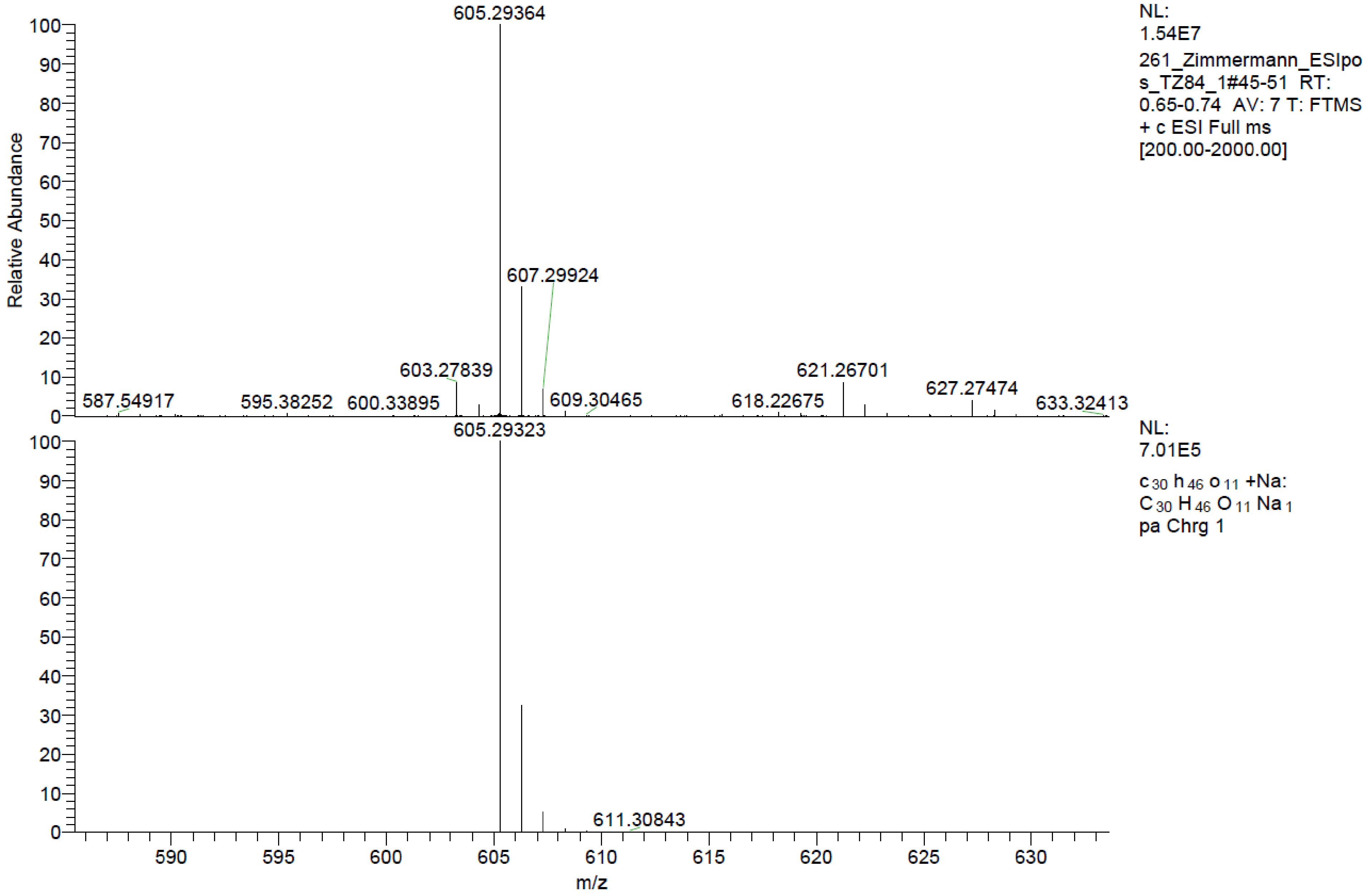

Figure S3D HRMS of product **3 -** $[C_{30}H_{46}O_{11}+Na]^+$ = 605.29364

Product **4** (8-*O*-((2*S*)-Methylbutanoyl)-8-*O*-debutanoyl thapsigargin)

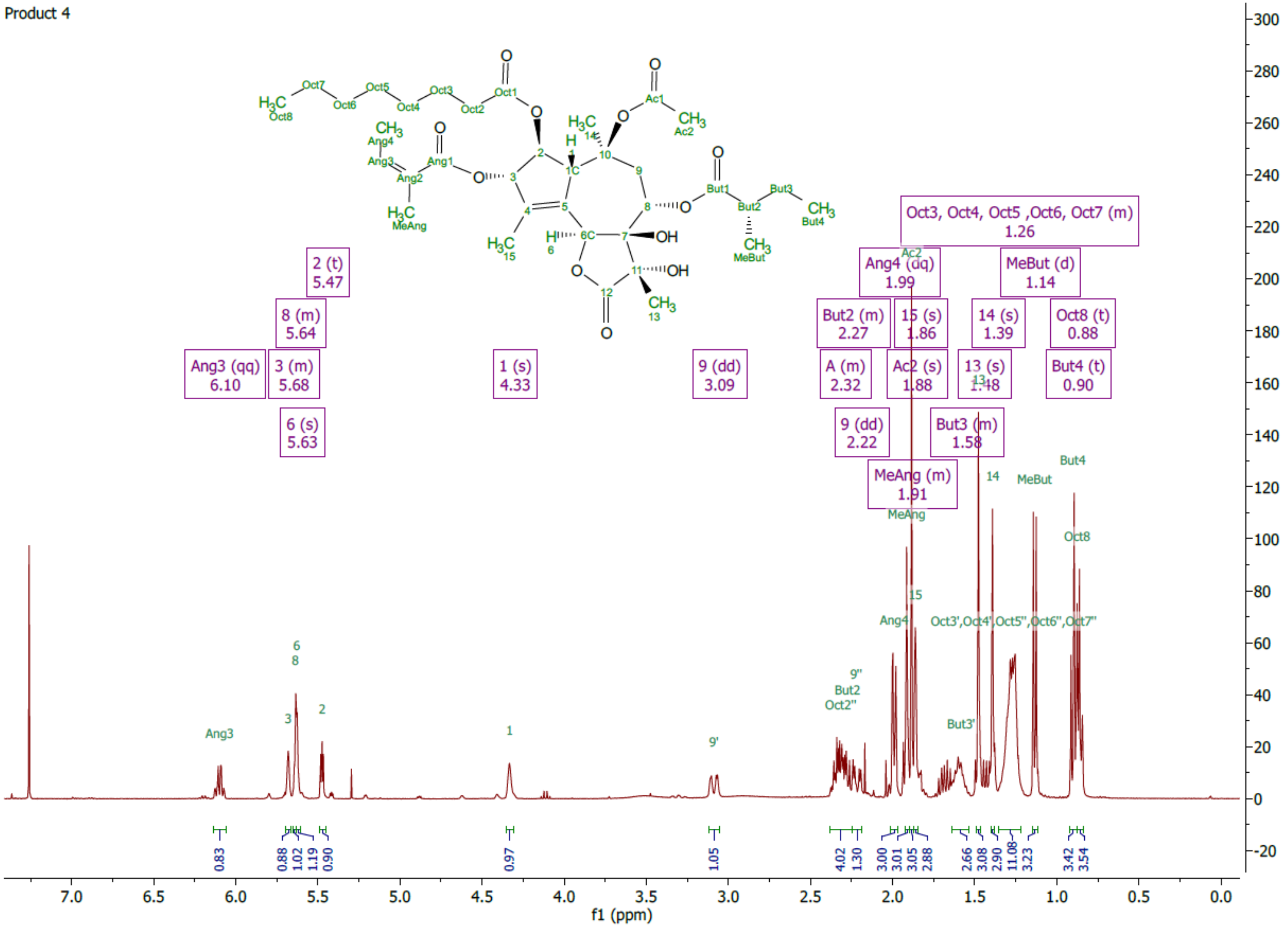

Figure S4A ^{1}H NMR of product **4** (400 MHz, $CDCl_3$)

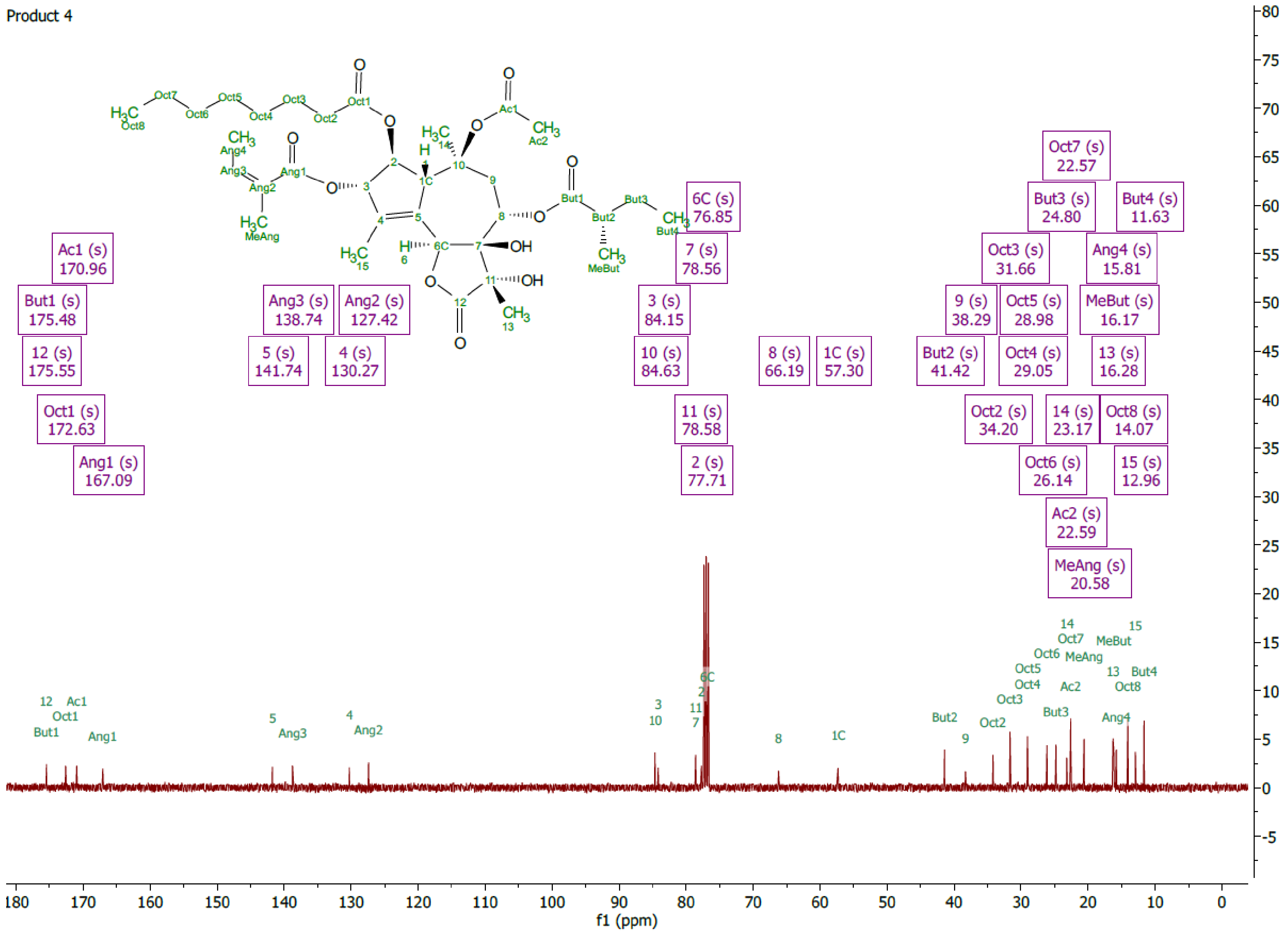

Figure S4B ^{13}C NMR of product **4** (100 MHz, $CDCl_3$)

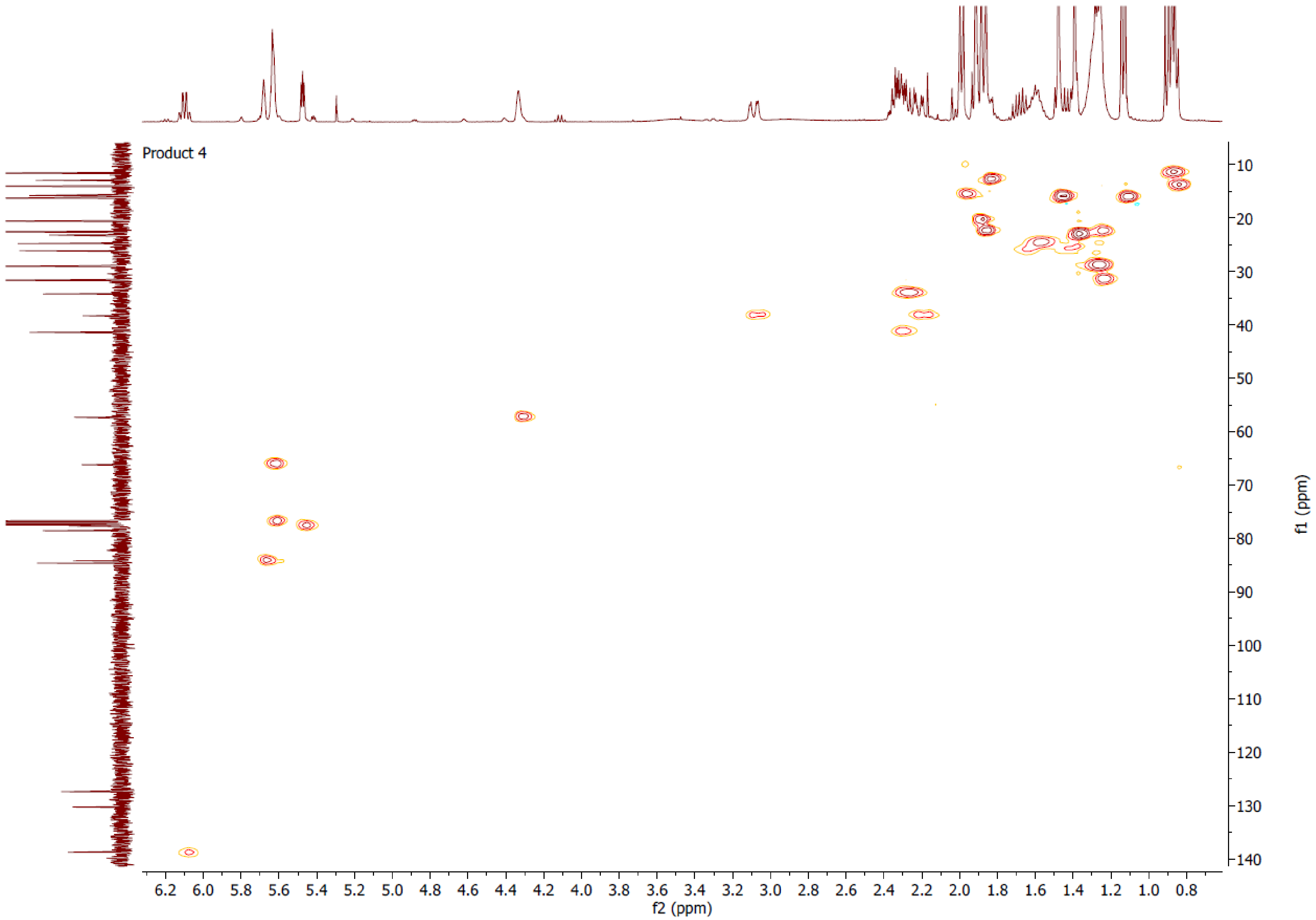

Figure S4C HMQC of product **4**

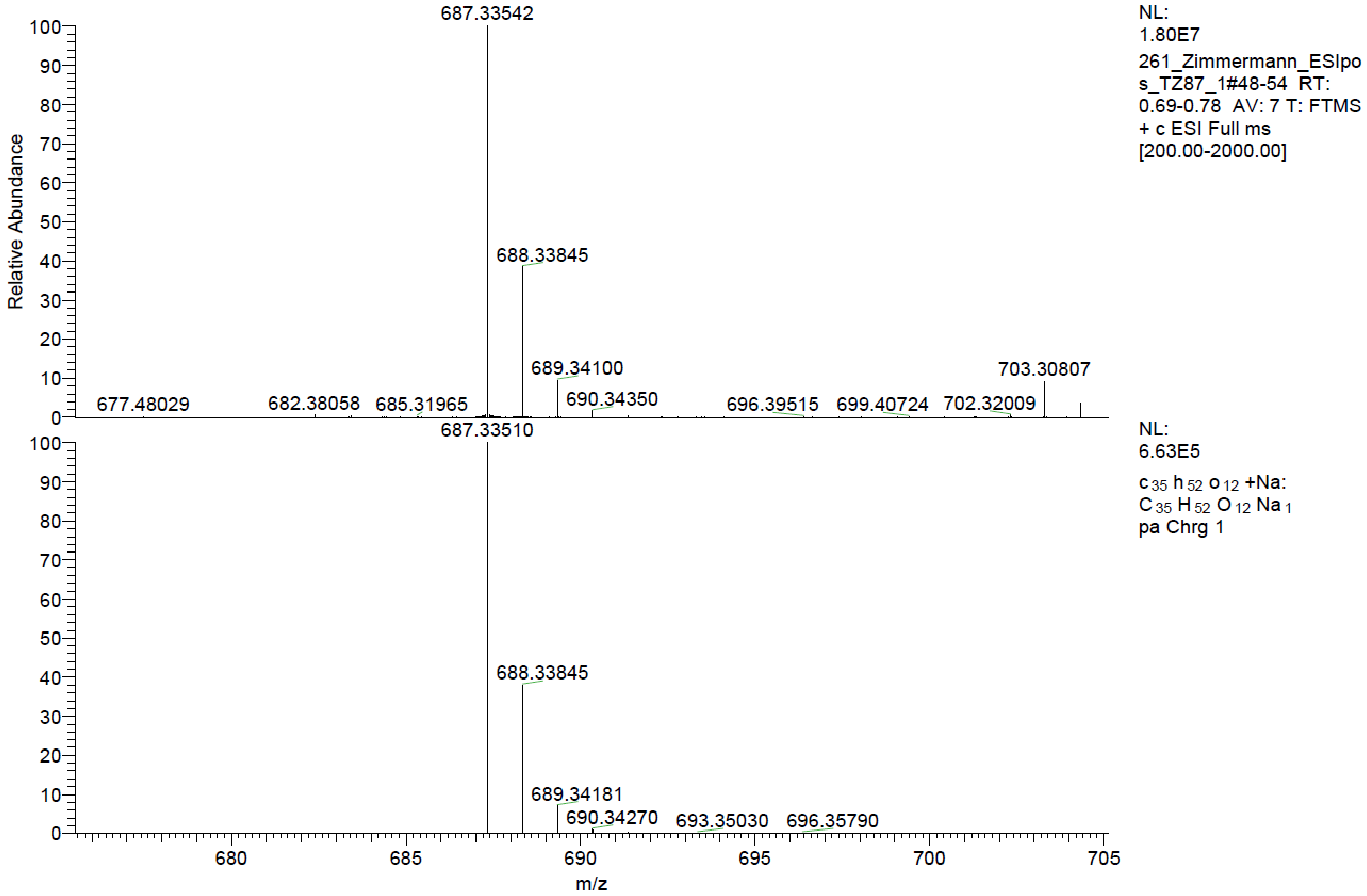

Figure S4D HRMS of product **4** **-** [$C_{35}H_{52}O_{12}$+Na]$^+$ = 687.33542

Product **5** (8-*O*-debutanoyl thapsigargin)

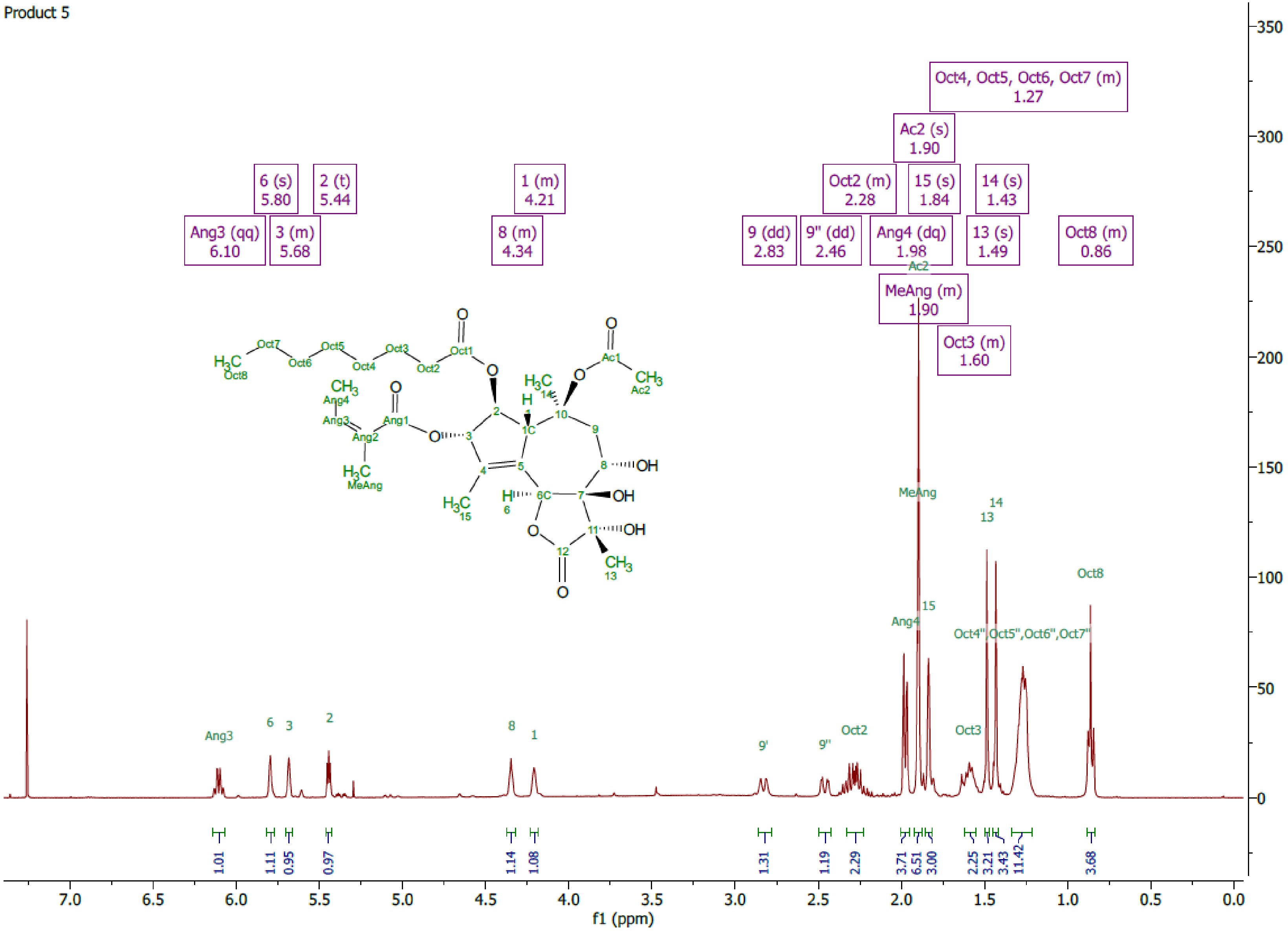

Figure S5A ^{1}H NMR of product **4** (400 MHz, $CDCl_3$)

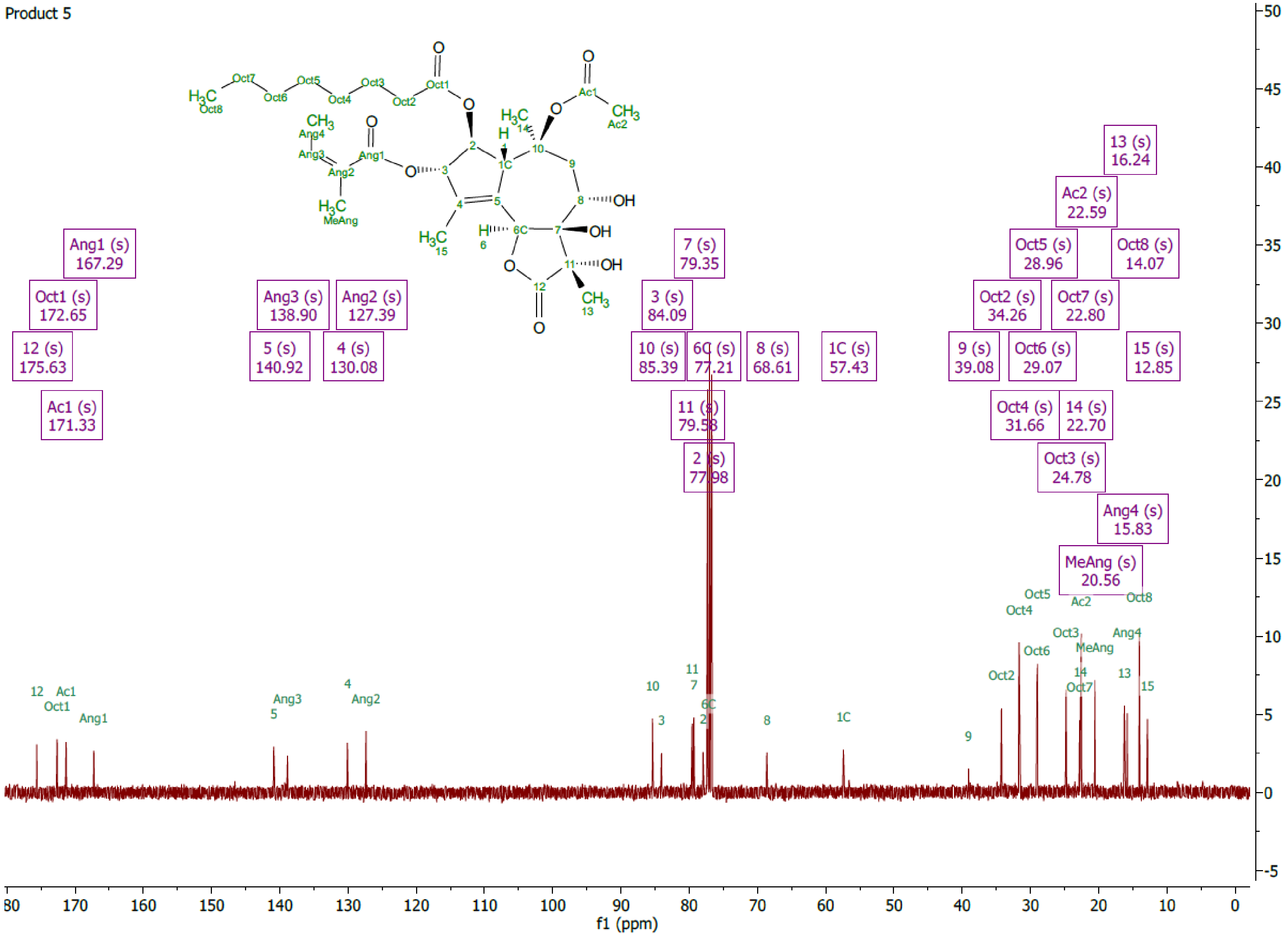

Figure S5B ^{13}C NMR of product **5** (100 MHz, $CDCl_3$)

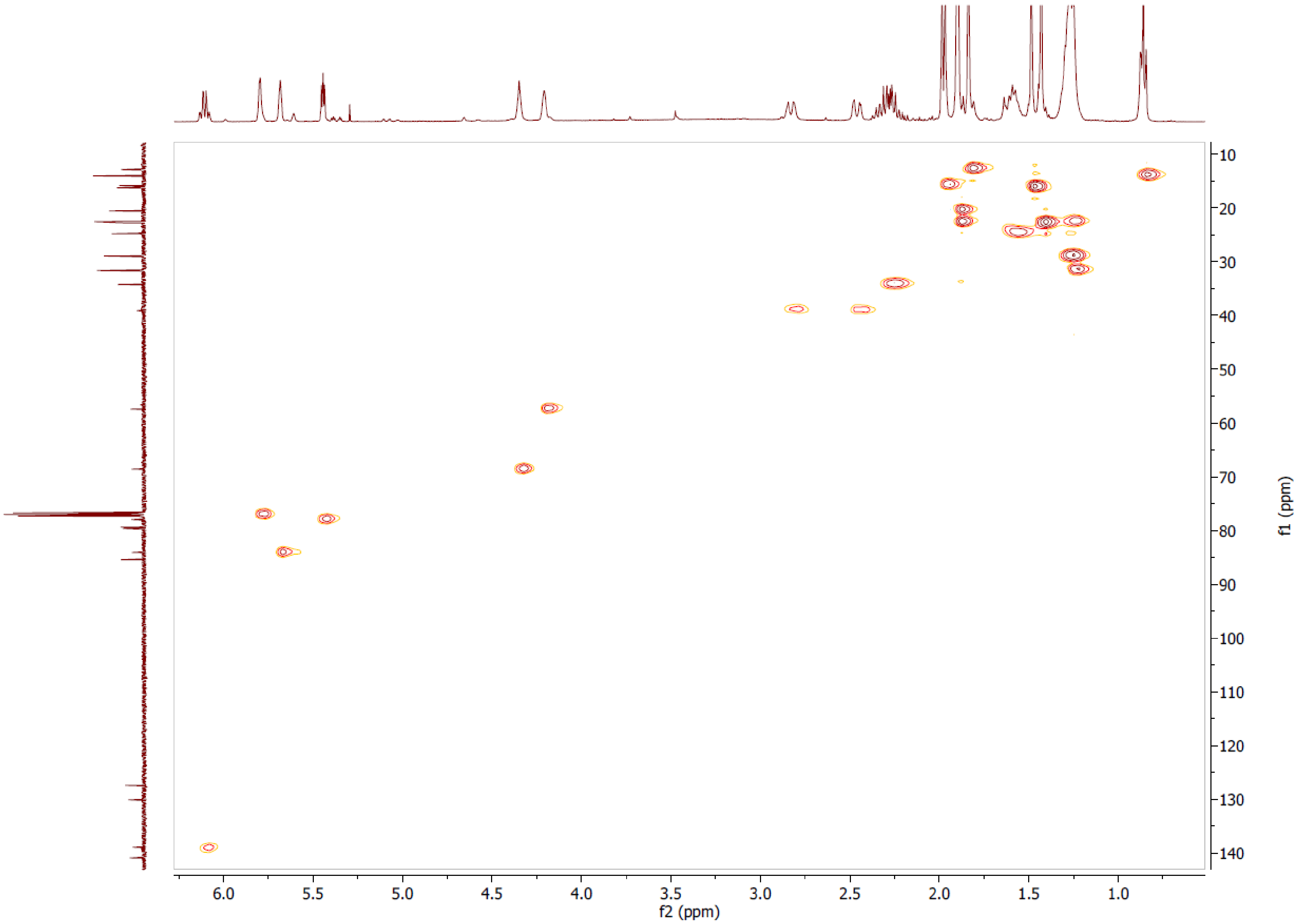

Figure S5C HMQC of product **5**

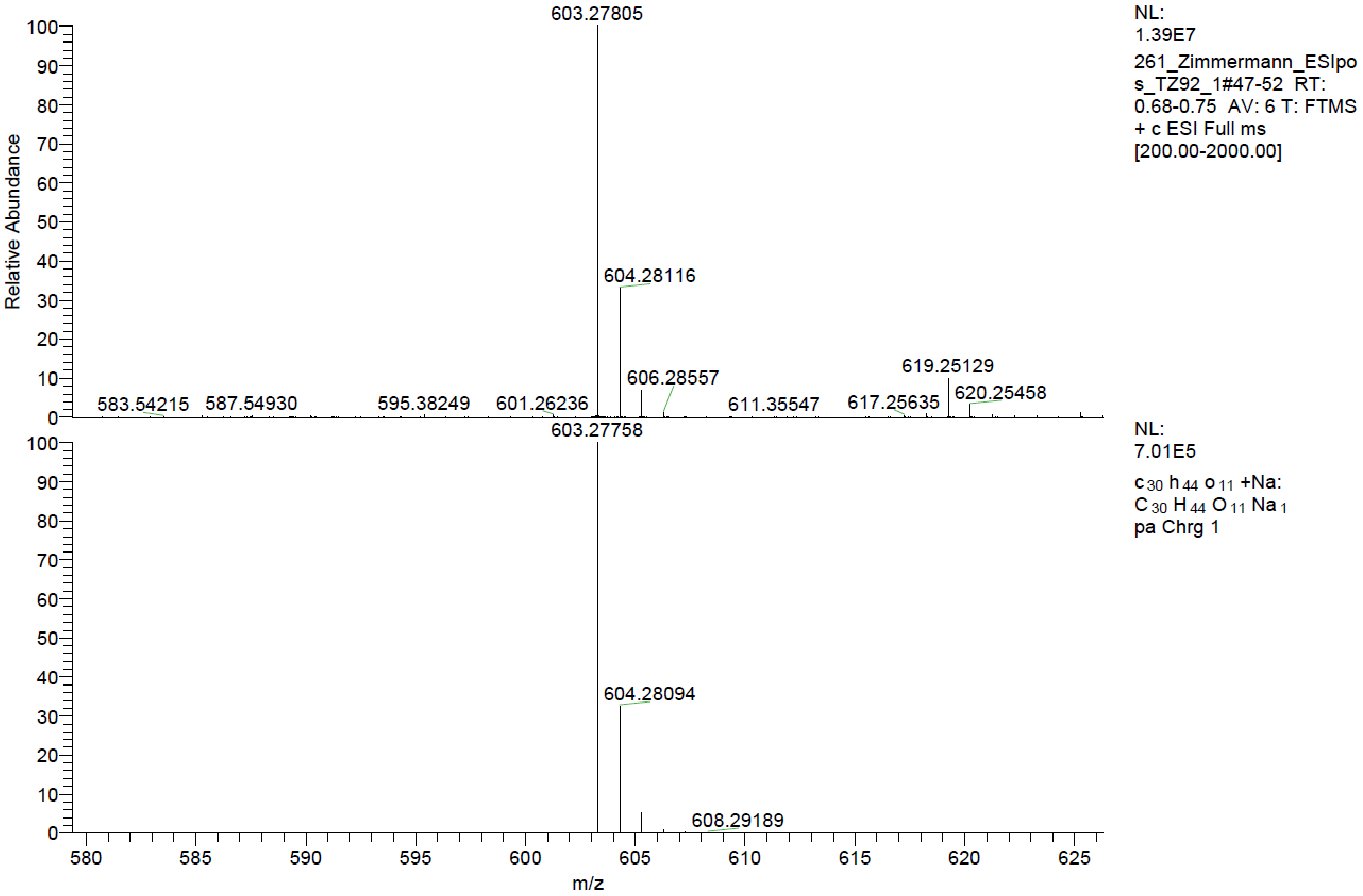

Figure S5D HRMS of product **5 -** $[C_{30}H_{44}O_{11}+Na]^{+}$ = 603.27805

Product **6** (8-*O*-(Boc-12-aminododecanoyl)-8-*O*-debutanoyl thapsigargin)

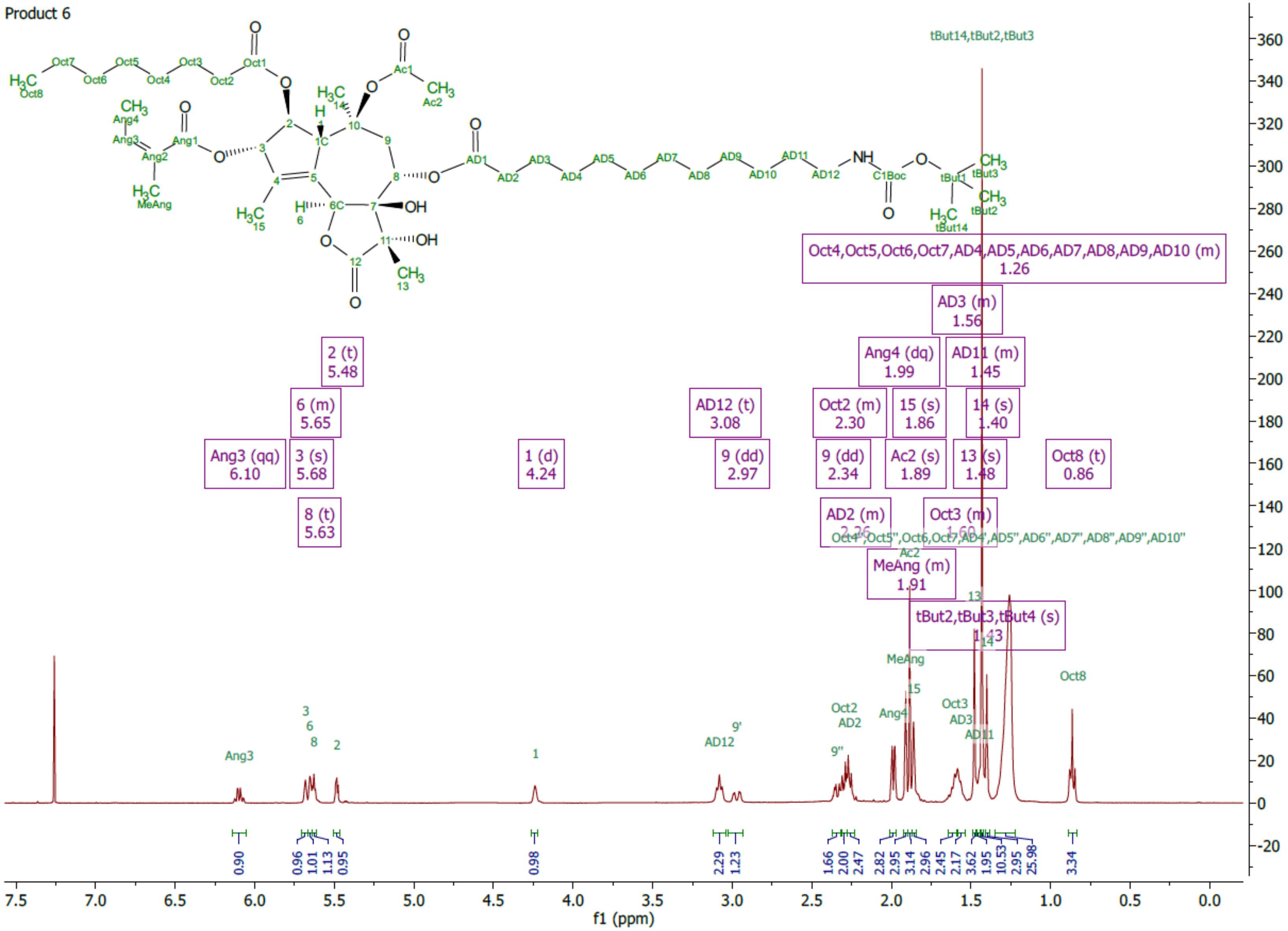

Figure S6A ^{1}H NMR of product **6** (400 MHz, $CDCl_3$)

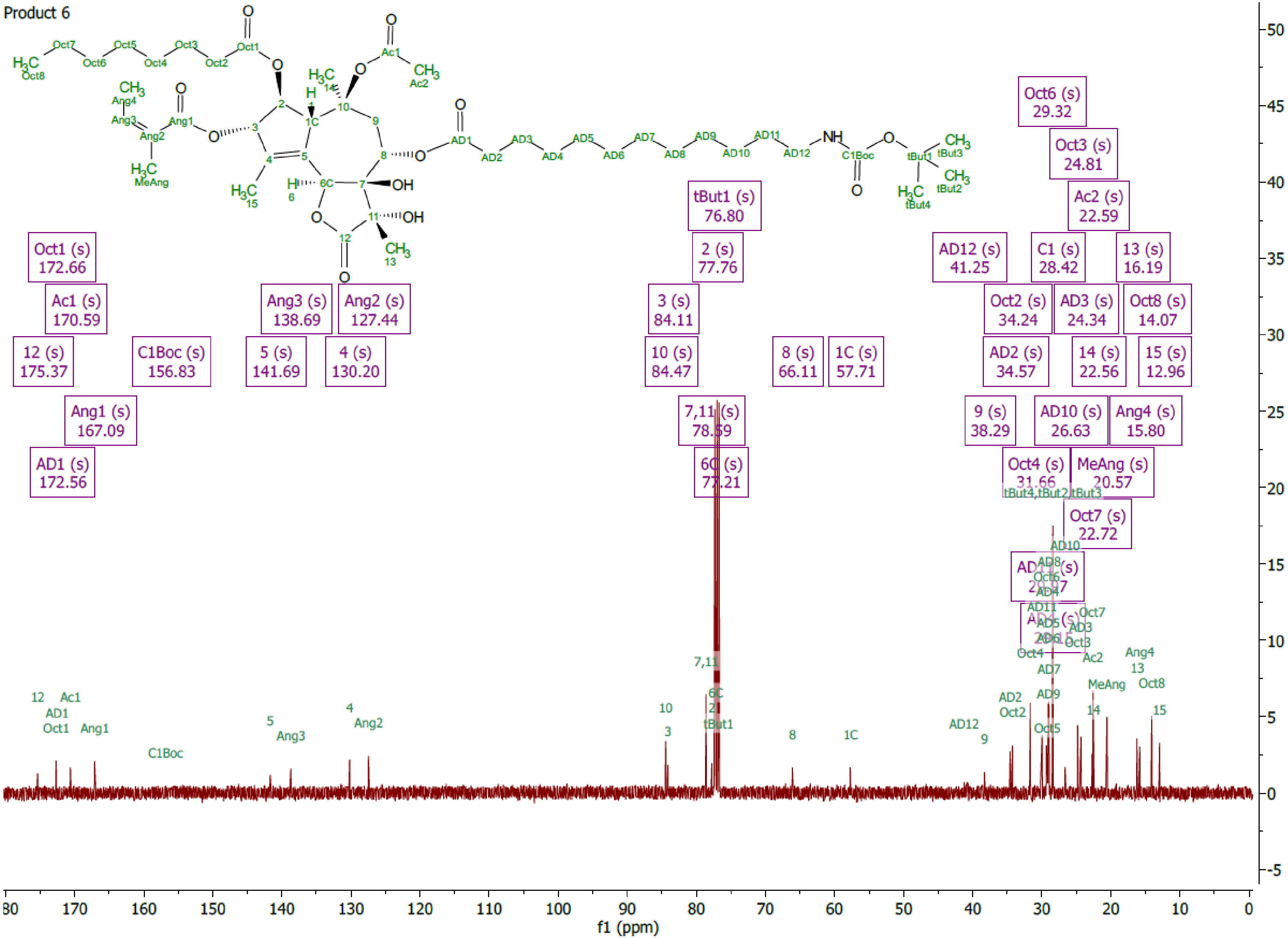

Figure S6B ^{13}C NMR of product **6** (100 MHz, $CDCl_3$)

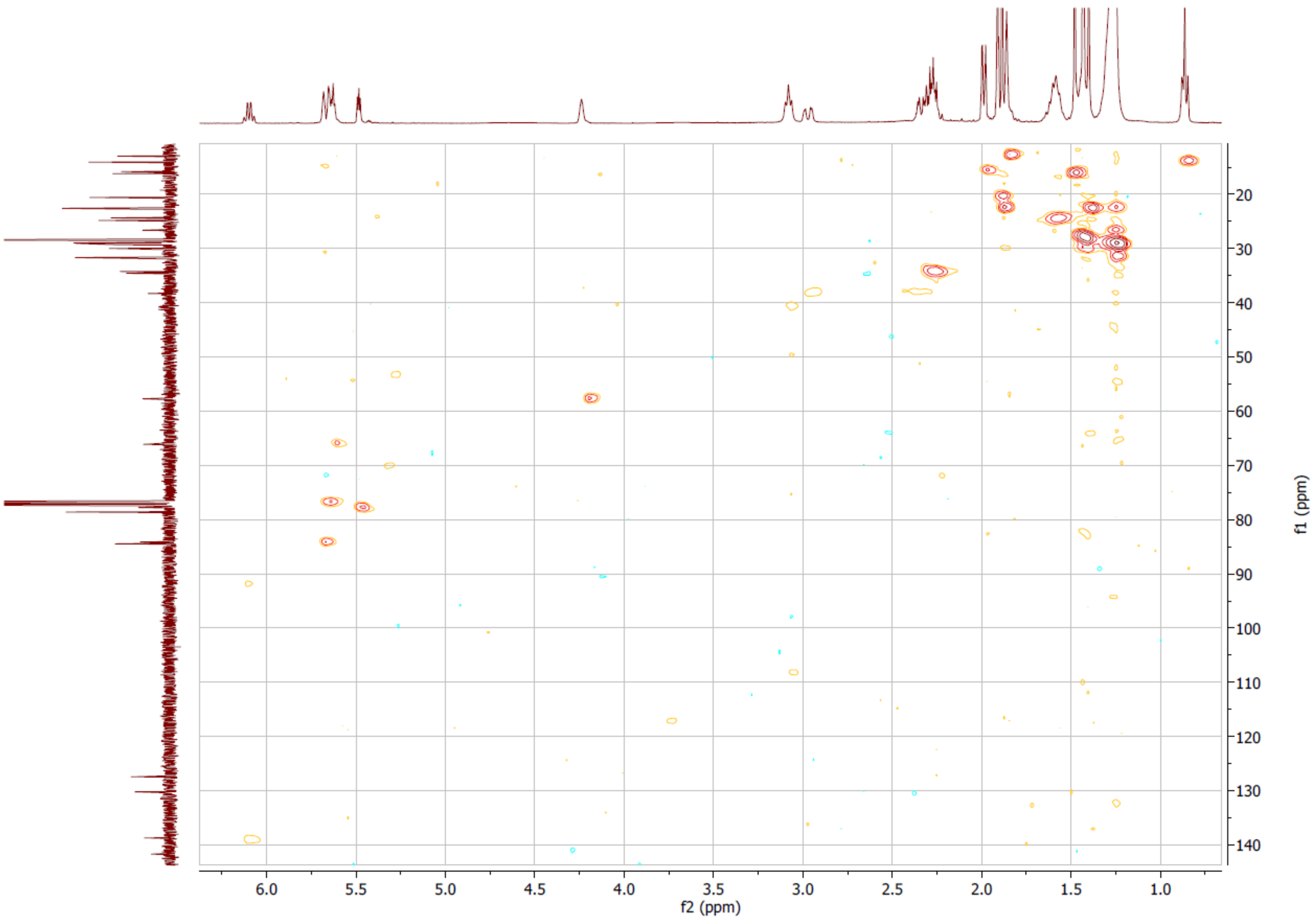

Figure S6C HMQC of product **6**

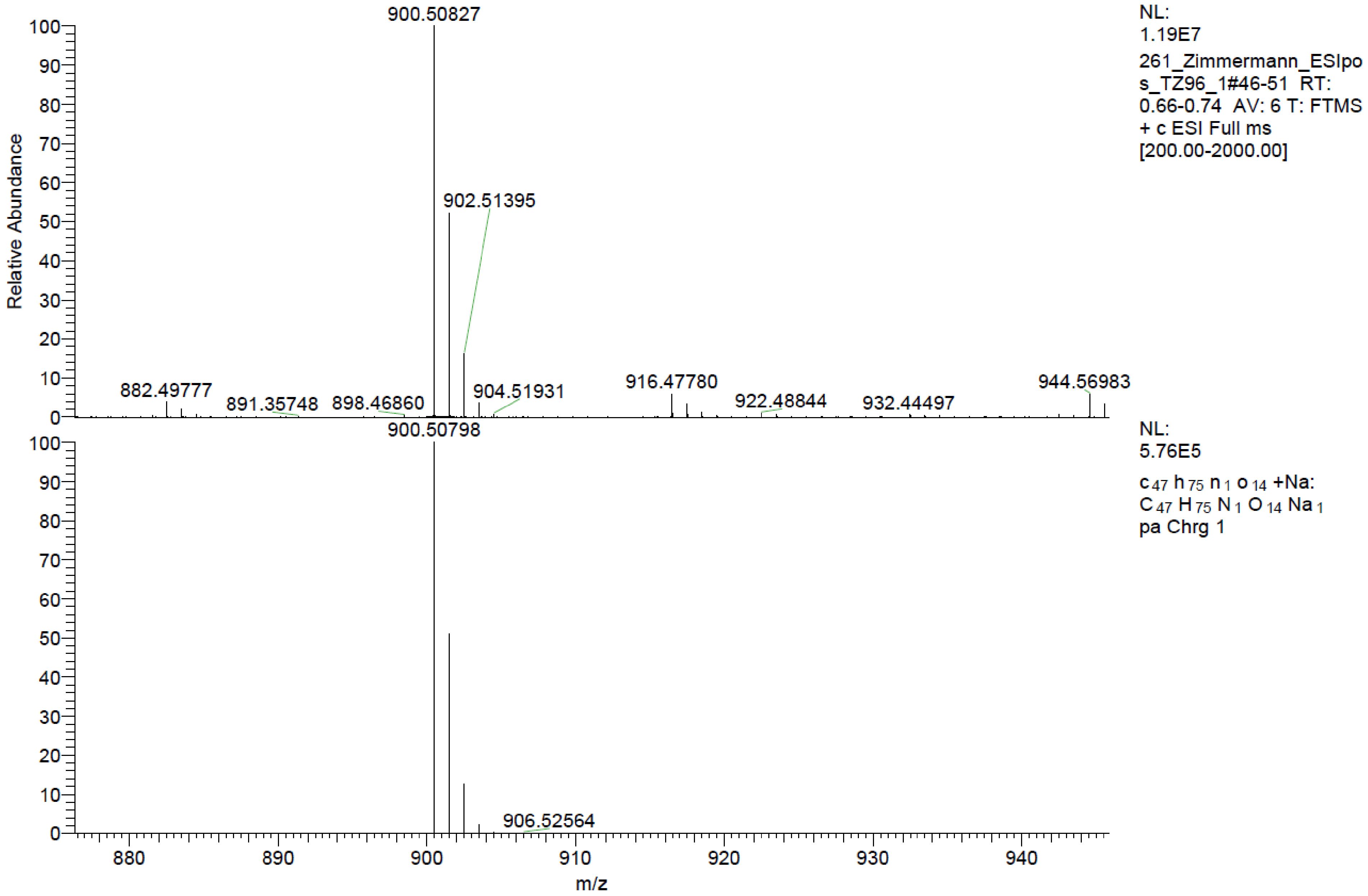

Figure S6D HRMS of product **6** - $[C_{47}H_{75}NO_{14}+Na]^+$ = 900.50827

Product **7** (8-*O*-(12-Aminododecanoyl)-8-*O*-debutanoyl thapsigargin)

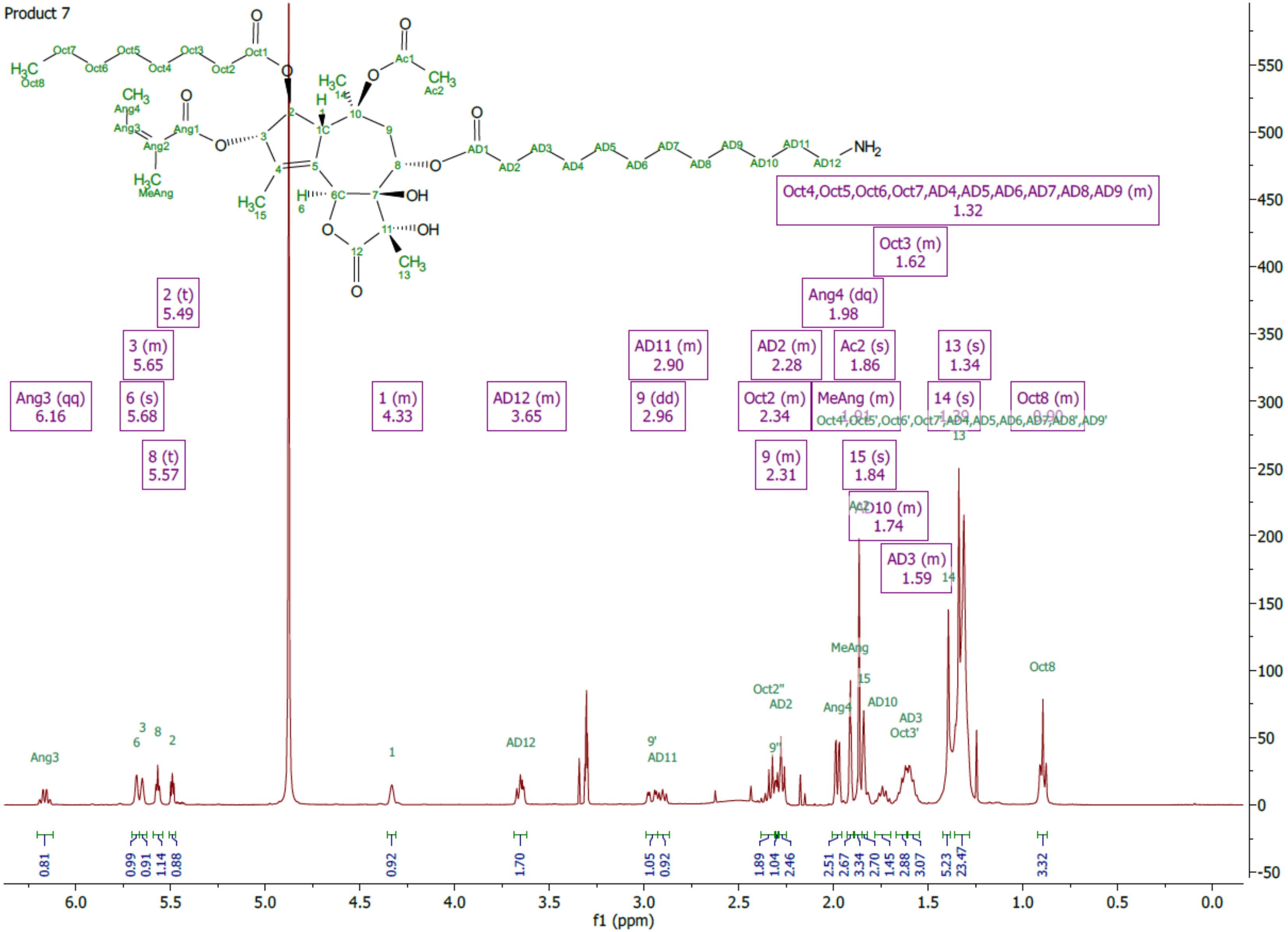

Figure S7A ^{1}H NMR of product **7** (400 MHz, CD_3OD)

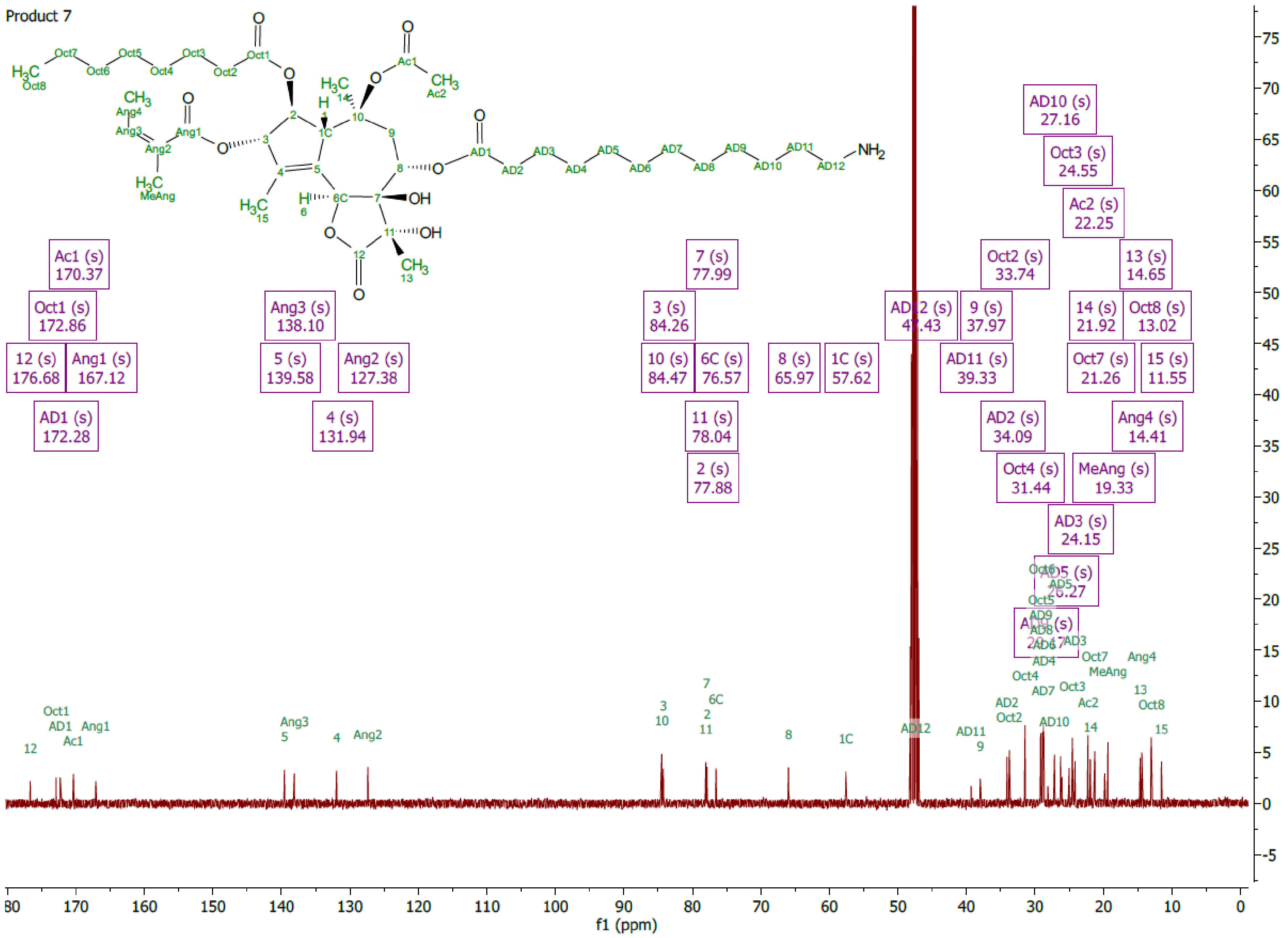

Figure S7B ^{13}C NMR of product **7** (100 MHz, CD_3OD)

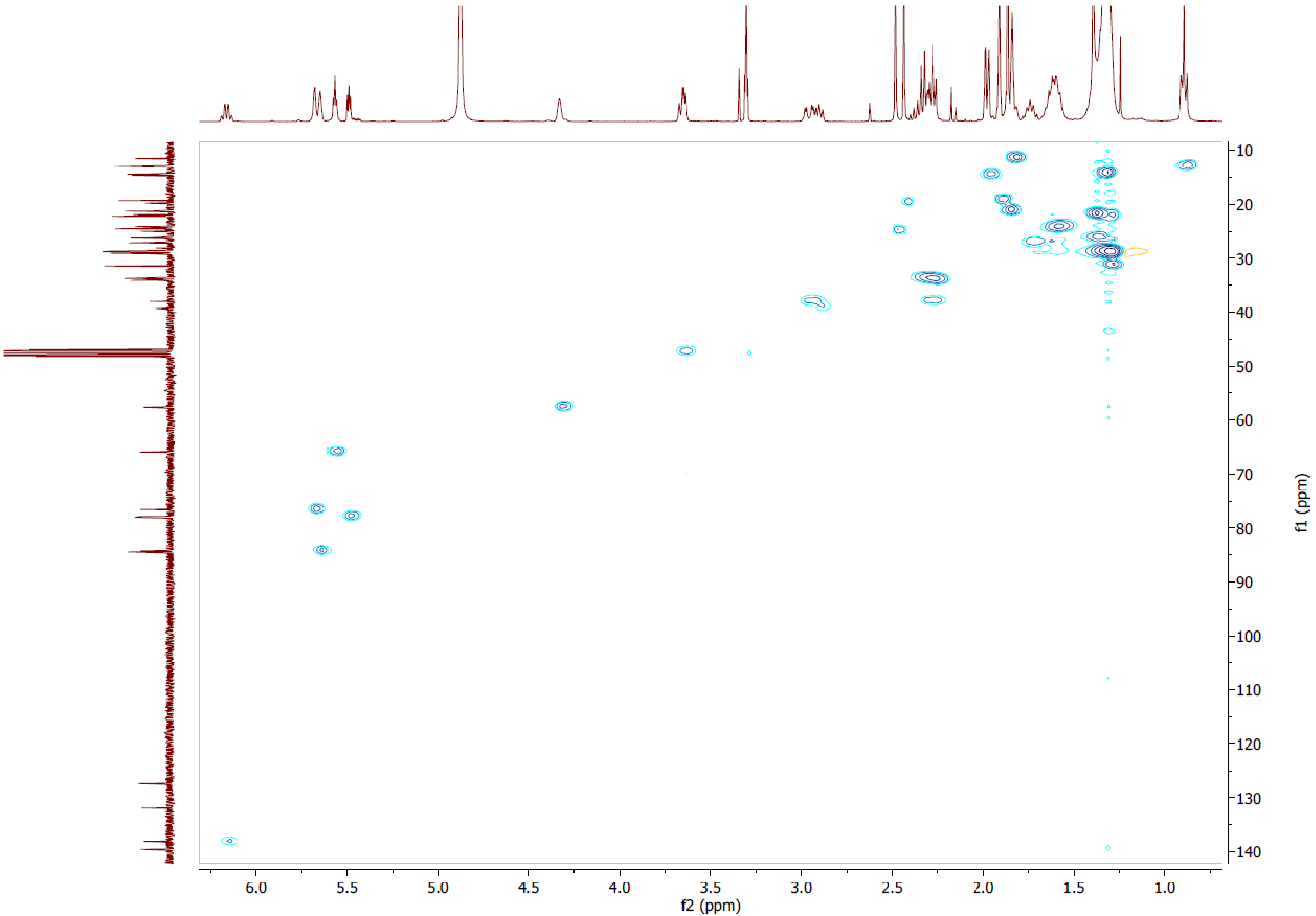

Figure S7C HMQC of product **7**

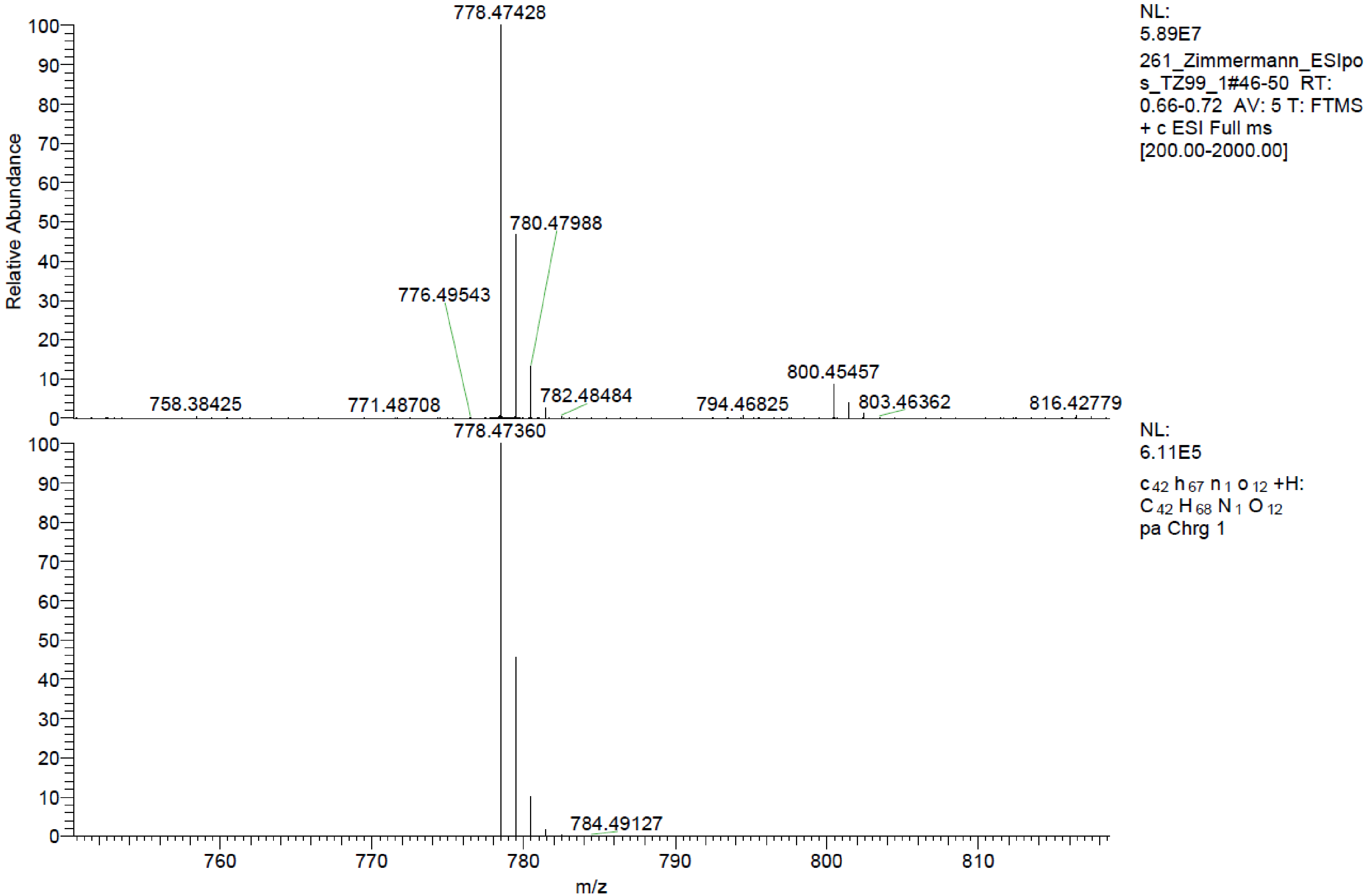

Figure S7D HRMS of product **7 -** $[C_{42}H_{67}NO_{12}+H]^{+}$ =778.47428

2,4,6-trichlorobenzoic (Z)-2-methylbut-2-enoic anhydride (**8**)

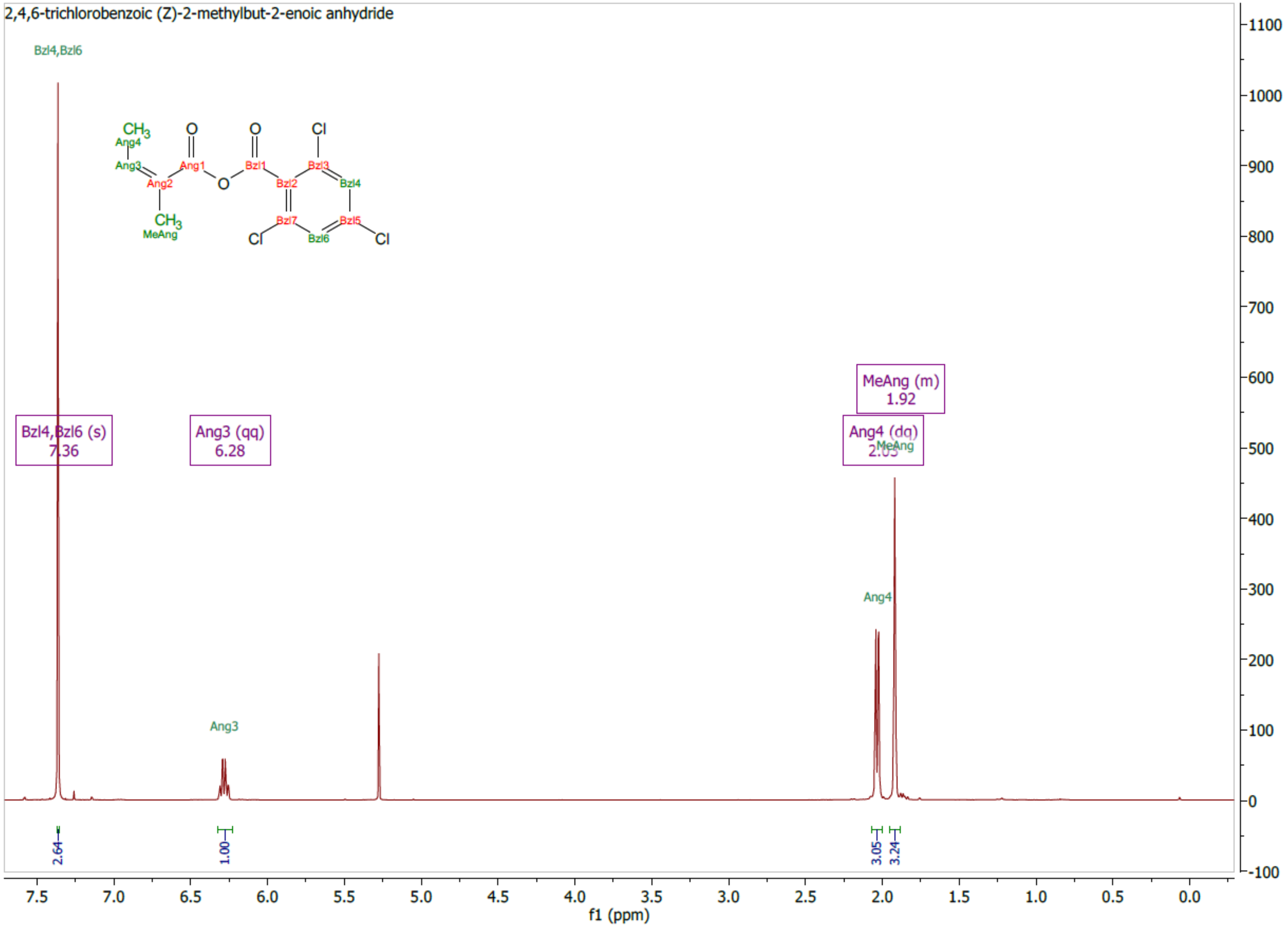

Figure S8A ^{1}H NMR of product **8** (400 MHz, CD_3Cl)

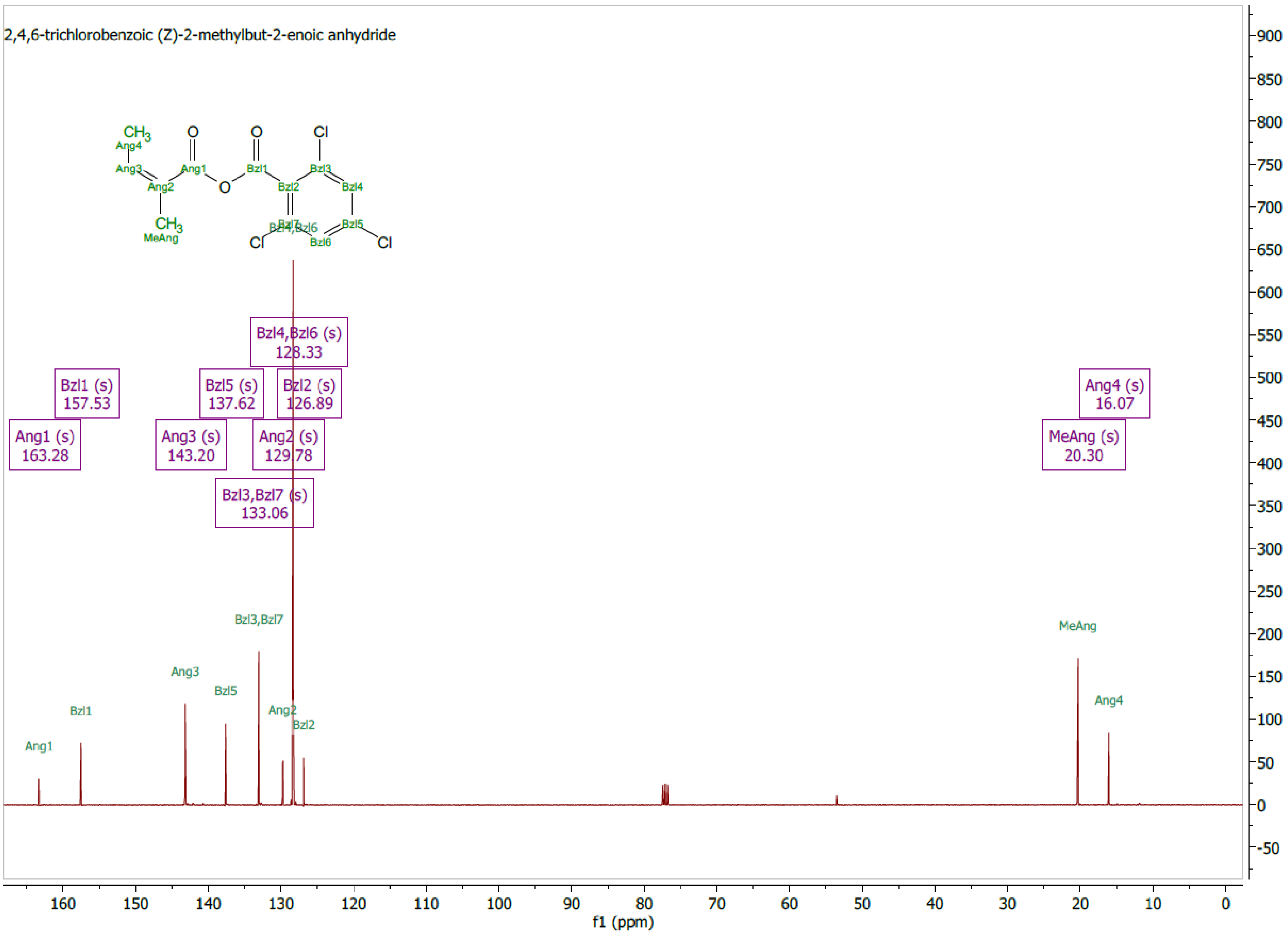

Figure S8B ^{13}C NMR of product **8** (100 MHz, CD_3Cl)

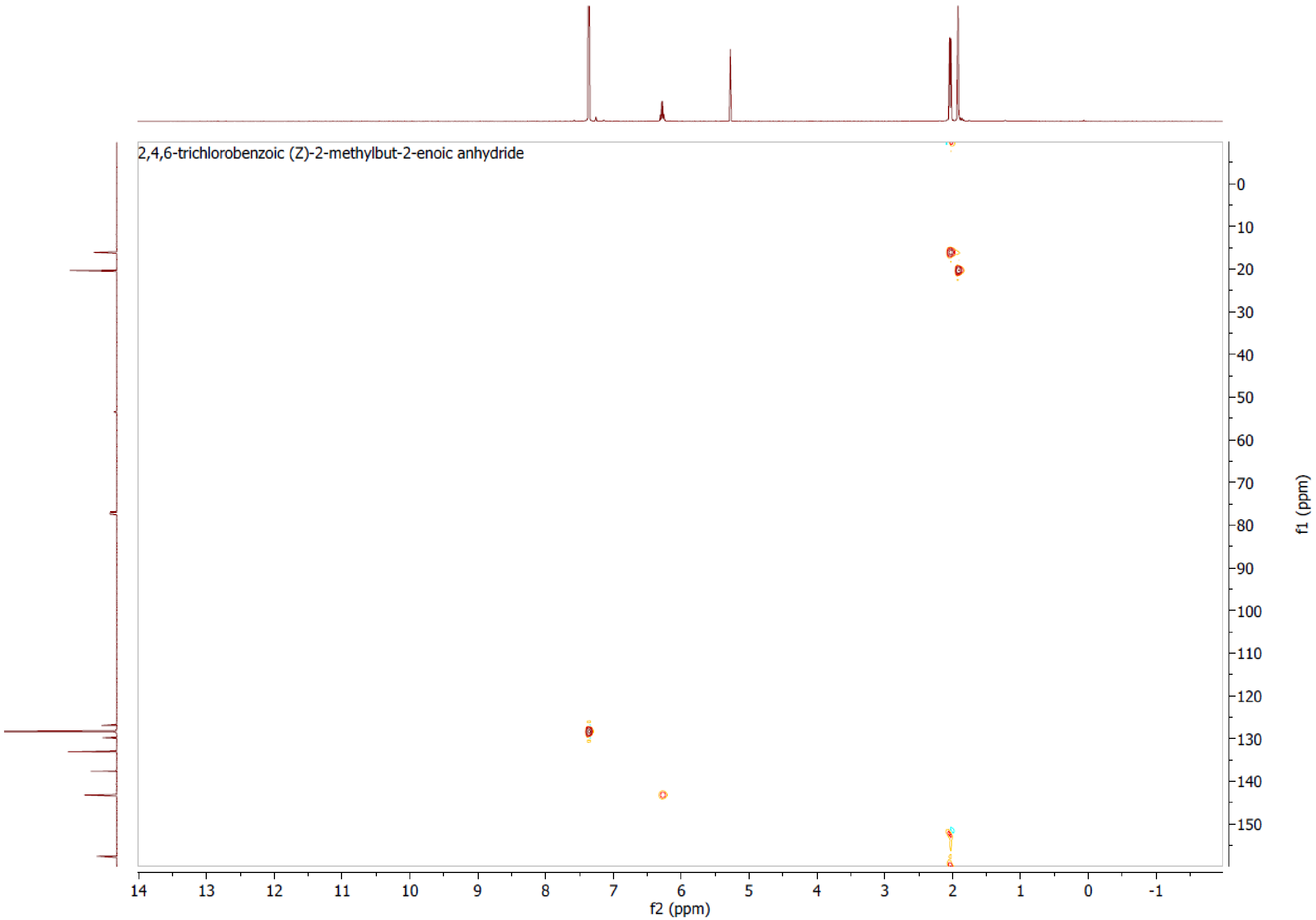

Figure S8C HMQC of product **8**

Boc-*N*-12-aminododecanoic acid (**9**)

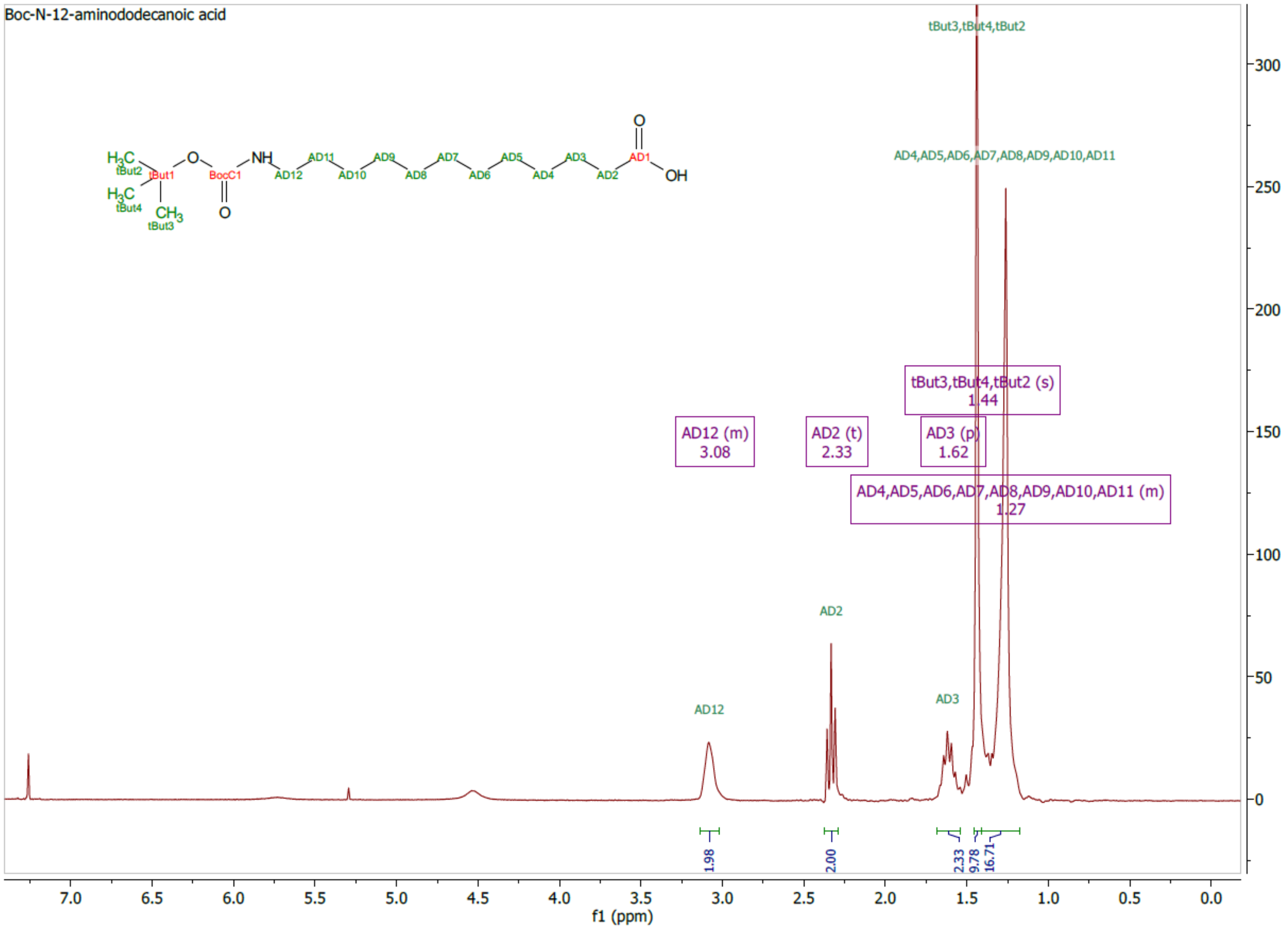

Figure S9A ^{1}H NMR of product **9** (400 MHz, CD_3Cl)

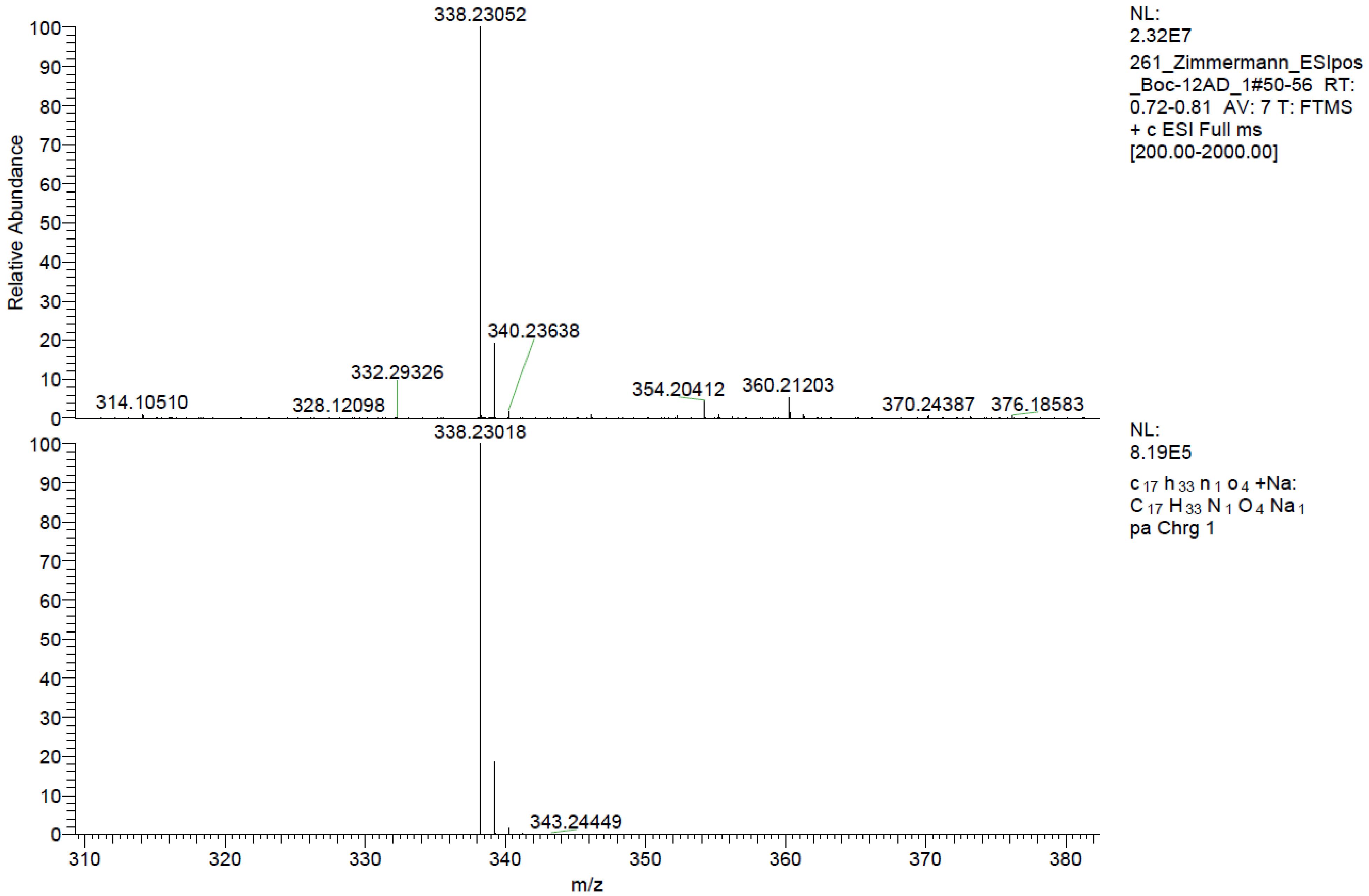

Figure S9B HRMS of product **9 -** $[C_{17}H_{33}NO_4+Na]^+ = 338.23052$

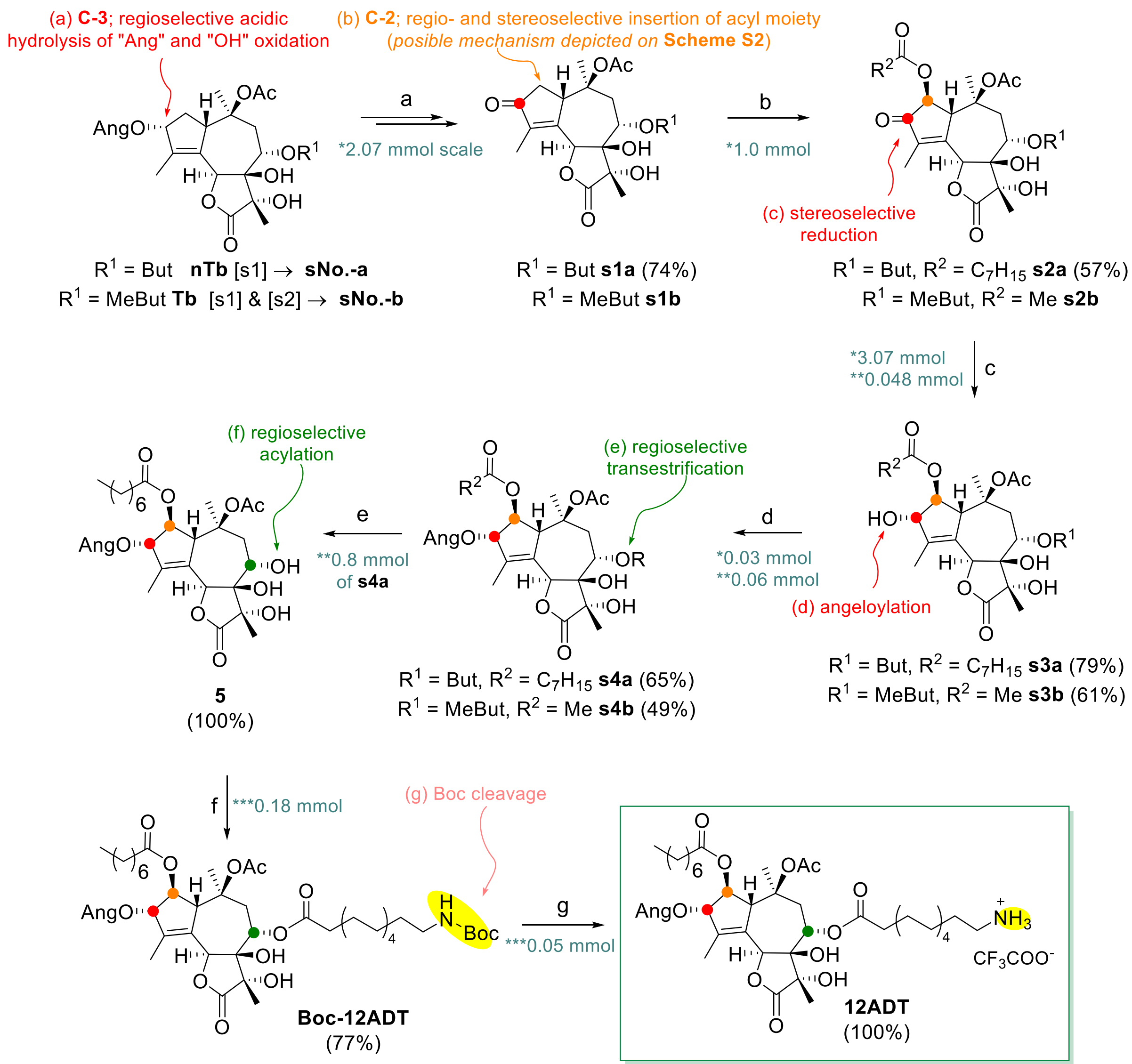

Scheme S1. The reactions addopted from the literature published [s1*, s2**, s3***]. *Reagents and conditions:* a) CrO_3, HF, AcCN, MW-95 °C, 2 h; b) octanoic acid, $Mn(OAc)_3 \cdot 2H_2O$, 120 °C, 6 h; c) $Zn(BH_4)_2$, Et_2O, -20 °C, 3.5 h, then H_2O; d) *in situ preparation of* 2,4,6-trichlorobenzoic (*Z*)-2-methylbut-2-enoic anhydride = acyl chloride, AngOH, TEA, in toluene; 90 °C, 18 h; e) TEA, MeOH, 0 °C→ 20 °C, 21 h; f) Boc-12-aminododecanoic acid, DCC, 4-DMAP, DCM, RT, 0 °C → RT, 6 h; g) TFA, DCM, H_2O, RT, 45 min. Under the arrows, the scale of the starting material is specified in green.

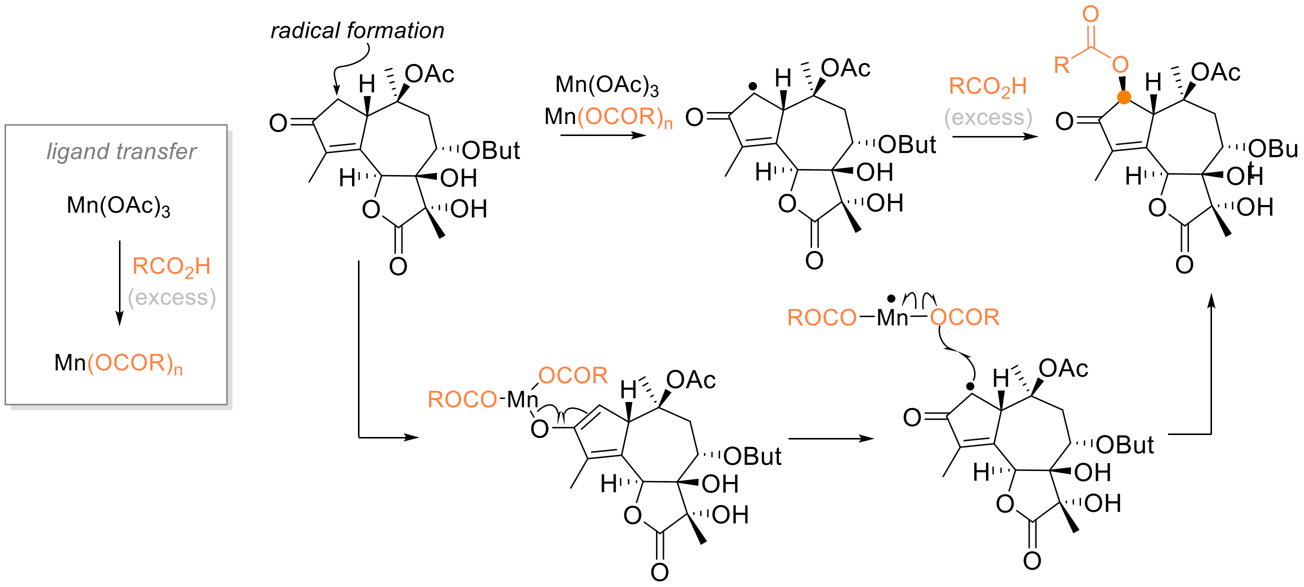

Scheme S2. Proposed mechanism of α-oxylation

References

[s1] Crestey, F.; Toma, M.; Christensen, S. B. Concise synthesis of thapsigargin from nortrilobolide. *Tetrahedron Lett.* **2015**, *56* (43), 5896-5898.

[s2] Doan, N. T. Q.; Crestey, F.; Olsen, C. E.; Christensen, S. B. Chemo- and regioselective functionalization of nortrilobolide: Application for semisynthesis of the natural product 2-acetoxytrilobolide. *J. Nat. Prod.* **2015**, 78 (6), 1406-1414.

[s3] Jakobsen, C. M.; Denmeade, S. R.; Isaacs, J. T.; Gady, A.; Olsen, C. E.; Christensen, S. B. Design, synthesis, and pharmacological evaluation of thapsigargin analogues for targeting apoptosis to prostatic cancer cells. *J. Med. Chem.* **2001**, *44*, 4696-4703.
